# The systemic cellular immune response against allogeneic mesenchymal stem cells is influenced by inflammation, differentiation and MHC compatibility: *in vivo* study in the horse

**DOI:** 10.3389/fvets.2024.1391872

**Published:** 2024-06-18

**Authors:** Alina Cequier, Francisco José Vázquez, Arantza Vitoria, Elvira Bernad, Sara Fuente, María Belén Serrano, María Pilar Zaragoza, Antonio Romero, Clementina Rodellar, Laura Barrachina

**Affiliations:** ^1^Biochemical Genetics Laboratory LAGENBIO, Institute for Health Research Aragón (IIS), AgriFood Institute of Aragón (IA2), University of Zaragoza, Zaragoza, Spain; ^2^Equine Surgery and Medicine Service, Veterinary Hospital, University of Zaragoza, Zaragoza, Spain

**Keywords:** horse, allogeneic, flow cytometry, immune response, co-culture, major histocompatibility complex, repeated administration, haplotype

## Abstract

The effectiveness and safety of allogeneic mesenchymal stem/stromal cells (MSCs) can be affected by patient’s immune recognition. Thus, MSC immunogenicity and their immunomodulatory properties are crucial aspects for therapy. Immune responses after allogeneic MSC administration have been reported in different species, including equine. Interactions of allogenic MSCs with the recipient’s immune system can be influenced by factors like matching or mismatching for the major histocompatibility complex (MHC) between donor-recipient, and by the levels of MHC expression in MSCs. The latter can vary upon MSC inflammatory exposure or differentiation, such as chondrogenic induction, making both priming and differentiation interesting therapeutic strategies. This study investigated the systemic *in vivo* immune cellular response against allogeneic equine MSCs in these situations. Either MSCs in basal conditions (MSC-naïve), pro-inflammatory primed (MSC-primed) or chondrogenically differentiated (MSC-chondro) were repeatedly administered subcutaneously into autologous, MHC-matched or MHC-mismatched allogeneic equine recipients. At different time-points after each administration, lymphocytes were obtained from recipient horses and exposed *in vitro* to the same type of MSCs to assess the proliferative response of different T cell subsets (cytotoxic, helper, regulatory), B cells, and interferon gamma (IFNγ) secretion. Higher proliferative response of helper and cytotoxic T lymphocytes and IFNγ secretion was observed in response to all types of MHC-mismatched MSCs over MHC-matched ones. MSC-primed produced the highest immune response, followed by MSC-naïve, and MSC-chondro. However, MSC-primed activated Treg and had a mild effect on B cells, and the response after their second administration was similar to the first one. On the other hand, both MSC-chondro and MSC-naïve barely induced Treg response but promoted B lymphocyte activation, and proportionally induced a higher cell response after the second administration. In conclusion, both the type of MSC conditioning and the MHC compatibility influenced systemic immune recognition of equine MSCs after single and repeated administrations, but the response was different. Selecting MHC-matched donors would be particularly recommended for MSC-primed and repeated MSC-naïve administrations. While MHC-mismatching in MSC-chondro would be less critical, B cell response should not be ignored. Comprehensively investigating the *in vivo* immune response against equine allogeneic MSCs is crucial for advancing veterinary cell therapies.

## 1 Introduction

Mesenchymal stem/stromal cells (MSCs) are adult multipotent stem cells that have attracted significant interest for regenerative medicine due to their distinct biological properties (1, 2). Since the underlying causes of certain diseases are similar in both people and animals, the findings obtained from MSC research can be valuable for both human and veterinary patients, promoting the concept of “One Health, One Medicine” (3, 4). Horses are of particular importance in translational medicine, as they suffer from various pathologies analogous to human with inflammatory and immune components, in which the ability of MSCs to modulate the immune system through their paracrine activity has broadened the scope of potential applications (5). The allogeneic application of MSCs in the treatment of these pathologies has several advantages over autologous therapy, including the possibility of making well-characterized MSCs more rapidly and widely available, especially when autologous cells are not suitable (6–8). Since there is now a broad consensus that MSCs are not truly immune-privileged, but rather immune-evasive, it is necessary to consider their recognition and elimination by the immune system in the allogeneic setting (9).

Allogeneic MSCs may trigger cellular and humoral immune responses, which can limit their therapeutic effects and potentially lead to adverse reactions (10, 11). Additionally, the development of immune memory mechanisms could influence the outcome of repeated administration of allogeneic MSCs (11, 12). Matching or mismatching for the major histocompatibility complex (MHC) could influence the recognition of the donor’s MSCs by the recipient’s immune system, but this factor is not always accounted when designing studies or clinical trials. In fact, MHC compatibility has been reported as a potential key factor for MSC therapy in several species, including human, horses and other animal models (8, 13–15). Actually, there is a growing interest in establishing haplobanks of human induced pluripotent stem cells (iPSCs) carrying the most common human leukocyte antigen (HLA) haplotypes (16), and similar strategies have also been used in equine MSC studies (10, 17). This approach involves selecting homozygous individuals as donors to enable compatibility with a wider range of heterozygous patients.

In addition to the MHC haplotype, MSC recognition is more or less likely depending on the expression level of MHC molecules in the surface of the cells. Importantly, the MHC expression level in equine MSCs can be intrinsic to the donor (13) but can also be modified by different factors, like inflammatory exposure and MSC differentiation (12, 18). Both factors are of particular importance as they represent situations that can occur *in vivo* after the administration of MSCs into the injury site, and that have also been studied as therapeutic strategies to enhance MSC effectiveness. Priming equine MSCs with pro-inflammatory cytokines like interferon gamma (IFNγ) and tumor necrosis factor alpha (TNFα) can promote their immunomodulatory properties, leading to improved regulatory effects (19, 20). However, this process can also raise their immunogenicity by increasing MHC expression (21, 22). In equine MSCs, low doses of IFNγ and TNFα during short priming periods allows maintaining a balance between the immunomodulatory and immunogenic profiles *in vitro* (23). On the other hand, chondrogenic differentiation of MSCs has been proposed as a therapeutic strategy not only to facilitate the integration of MSCs at the site of cartilage injury but also to stimulate the secretion of specific molecules potentially beneficial for joint pathologies (24). However, chondrogenic differentiation can decrease MSC immunomodulatory ability and increase their immunogenicity by raising *MHC-I* and/or *MHC-II* expression (25), so MSCs could lose their particular immune properties when becoming a specialized cell type (18, 26).

In spite of important advancement in our understanding about the interactions between MSCs and the immune system, the clinical implications of the immune recognition of allogeneic MSCs remains to be fully elucidated. While *in vitro* studies offer key preliminary insight, these observations do not always translate into the *in vivo* scenario, where the administration of allogeneic MSCs has generally demonstrated to be safe (8, 10, 27, 28) but with variable effectiveness (29). This suggests a balance between the immunogenicity and the immunomodulatory properties of allogeneic MSCs that may determine to what extent they can evade the immune system and exert their therapeutic effects (21).

However, evaluating the immune properties of MSCs *in vivo* is challenging as only a small percentage of cells is retained at the site of administration (30). Encapsulating MSCs in alginate hydrogels is a common approach (31) that enables the interchange of nutrients and metabolites between the organism and the MSCs and maintains the latter in a definite location (18), thus constituting an advantageous setup for investigating their interaction with the recipient’s immune system (32). Such interactions can be studied by using modified one-way mixed lymphocytes reactions (MLRs). This system allows evaluating the response of the recipient’s lymphocytes (responder cells) when they are exposed *in vitro* to the same MSCs that were previously administered *in vivo* (stimulator cells) (6). Importantly, most of the previous research using co-cultures of equine MSCs and lymphocytes has focused on the MSC capacity to regulate exogenously activated lymphocytes (i.e., immunomodulation), while only a few have investigated the MSC potential to stimulate a response in resting lymphocytes (13, 33, 34).

To better understand the interactions of equine MSCs with the immune system *in vivo*, we aimed at assessing the effect of MSC pro-inflammatory priming, chondrogenic differentiation and donor-recipient MHC compatibility on the systemic immune cell response after single and repeated administration. To do this, we used a system of subcutaneous implantation of hydrogel-encapsulated equine MSCs in basal conditions (MSC-naïve), pro-inflammatory primed (MSC-primed) or chondrogenically differentiated (MSC-chondro) into MHC-matched/mismatched recipients. At different times after each administration, lymphocytes were obtained from recipient horses and exposed *in vitro* to the same MSCs previously received. By using this modified one-way MLR system, we assessed the proliferative response of relevant lymphocyte subsets and their production of IFNγ to understand the generation of immune responses *in vivo*.

Our hypothesis was that the immunomodulatory capacity induced in MSC-primed would allow them to evade the recipient’s immune system, while differentiated MSC-chondro might trigger a stronger immune response. In addition, we hypothesized that MHC incompatibility would further increase the immune reaction elicited by any type of MSCs. To the best of the authors’ knowledge, this is the first *in vivo* study that simultaneously investigates the effect of all these three factors (inflammation, chondrogenic differentiation, and MHC compatibility) on the administration of MSCs *in vivo*.

## 2 Materials and method

### 2.1 Study design

**Figure 1:**
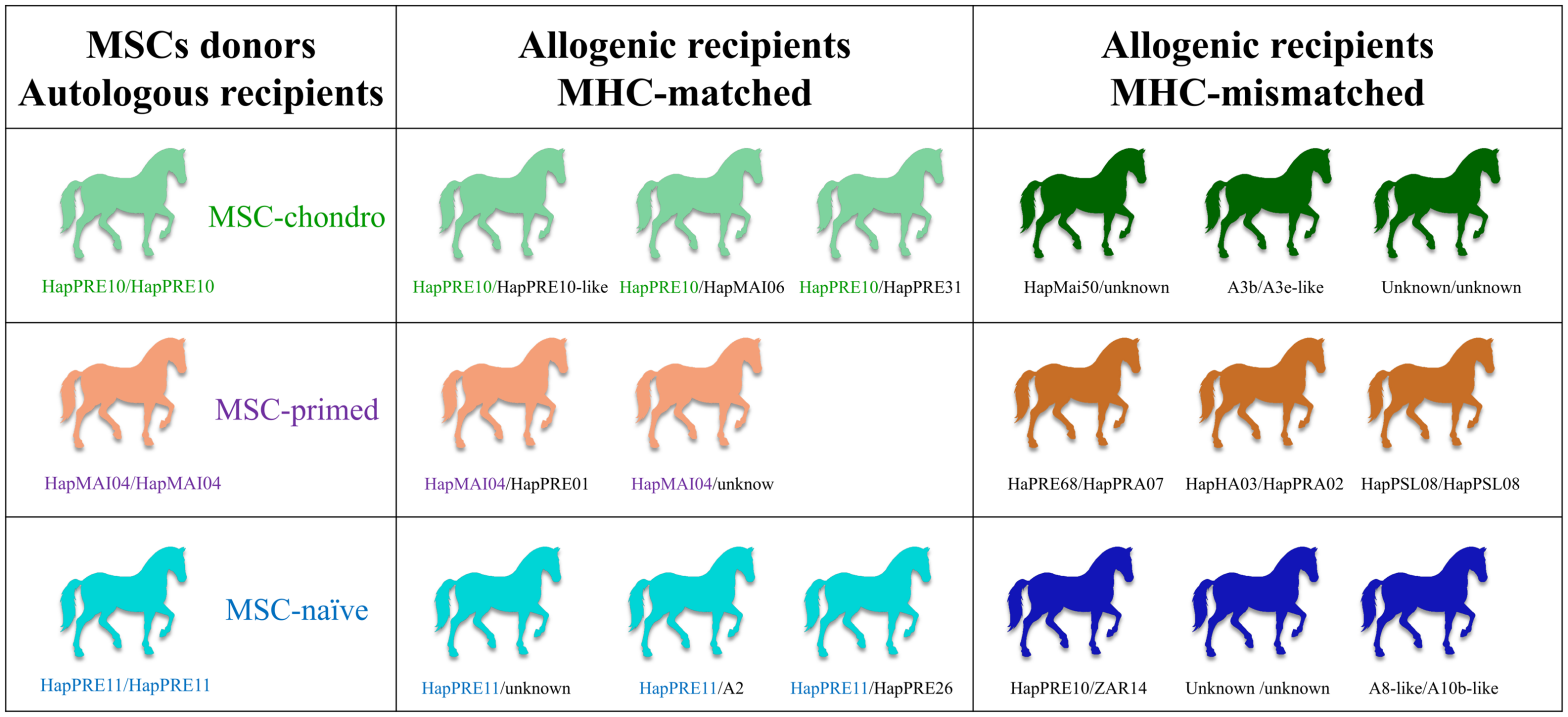
Study design. Groups of recipient horses (autologous—also acted as donors-, allogenic MHC-matched and allogenic MHC-mismatched) and types of MSCs received (MSC-chondro, MSC-primed and MSC-naïve). The MHC haplotypes of each individual are indicated. Supplementary material S1 shows the microsatellite alleles of each haplotype identified in the different horses.

Equine bone marrow derived MSCs (BM-MSCs) were administered twice to MHC-matched and MHC-mismatched recipient horses, and the systemic cellular immune response was serially analyzed using a modified one-way MLR system. Three horses homozygous for the MHC-haplotype (HapPRE10/HapPRE10, HapPRE11/HapPRE11, and HapMAI04/HapMAI04) were selected as MSC donors (17). The recipient horses were categorized into two groups: MHC-matched and MHC-mismatched with the donors. The MHC-matched group included eight heterozygous horses that shared one haplotype with one of the donors: three HapPRE10 heterozygotes, three HapPRE11 heterozygotes and two HapMAI04 heterozygotes. For the MHC-mismatched group, nine horses were selected, all of them carrying haplotypes other than HapPRE10, HapPRE11 and HapMAI04. Within each group of recipients (MHC-matched and MHC-mismatched), animals were arranged into three subgroups of 2–3 animals. Each subgroup received either basal MSCs (MSC-naïve), proinflammatory-primed MSCs (MSC-primed) or chondrogenically differentiated MSCs (MSC-chondro). In addition, homozygous donors (*n* = 3) acted as autologous controls receiving each one MSC-naïve, MSC-primed or MSC-chondro (Figure 1).

**Figure 2:**
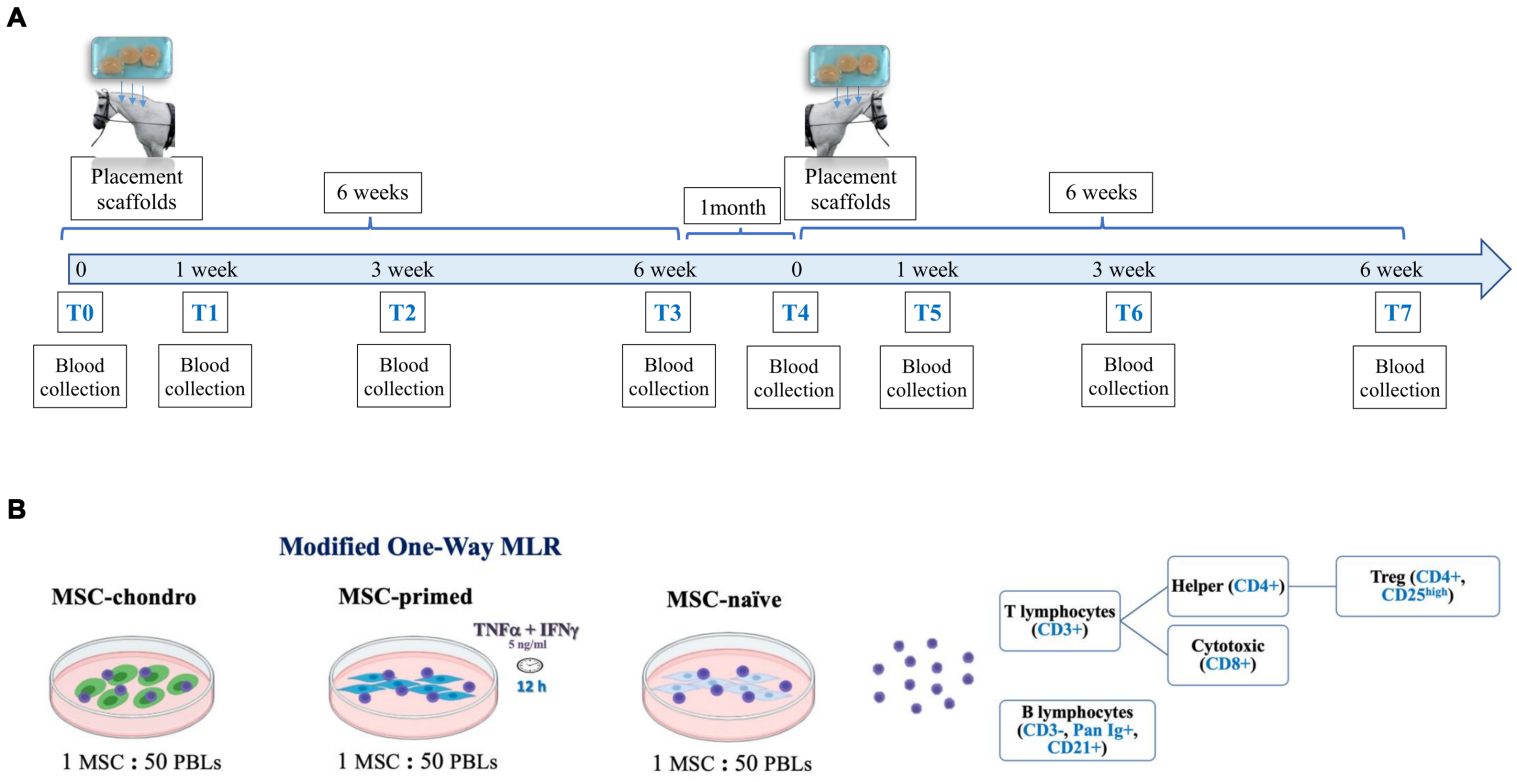
Schematic representation of the study design. **(A)** Three scaffolds containing the same type of MSCs were subcutaneously placed in either the donor horses (autologous recipients) or in MHC-matched or MHC-mismatched allogeneic recipient horses. Blood was collected at different time-points afterwards (1, 3 and 6 weeks) and, 1 month later, the procedure was repeated to evaluate the re-exposition to the same type of MSCs. **(B)** Modified one-way mixed lymphocyte reaction (MLR) assays were performed by co-culturing PBLs from the recipients with the same type of MSCs (MSC-naïve, MSC-primed, MSC-chondro: MHC-matched/mismatched) previously administered *in vivo*. After co-culture, PBLs and supernatant were collected to evaluate the frequency and proliferation of each lymphocyte subset by flow cytometry and the secretion of IFNγ by ELISA.

Each animal received simultaneously three alginate hydrogel scaffolds, each one containing 5 × 10^6^ MSCs of the corresponding type (MSC-naïve, MSC-primed or MSC-chondro). The scaffolds were placed in the neck by creating subcutaneous pockets. This approach was taken to retain the MSCs in a specific anatomic site and minimize interferences of their migration. In addition, this system allowed recovering the scaffolds to be used in a separate study assessing the local response (data not published). In the present study, peripheral blood was collected from each animal to obtain lymphocytes for the one-way MLR assays. This was done prior to scaffold placement (T0) and at weeks 1 (T1), 3 (T2) and 6 (T3) following the implantation. One month later, each horse was subjected to the same procedure to mimic the effect of a second administration: each animal received three new scaffolds in the contralateral neck side, containing the same type and dose of MSCs than in the first administration. Blood was collected at the same time-points (T4: prior to MSC re-exposure; T5: 1 week after the re-exposure; T6: 3 weeks after the re-exposure and T7: 6 weeks after the re-exposure). Schematic timeline and methods are shown in Figure 2A.

A modified one-way MLR assay was used to evaluate the cellular immune response at the systemic level and the possible development of cellular-mediated immune memory. Following blood collection, peripheral blood lymphocytes (PBLs) were obtained from all the recipients and at all the time-points indicated. The PBLs of each recipient were re-exposed *in vitro* to the same type of MSCs that were previously administered *in vivo* (MSC-naïve, MSC-primed, MSC-chondro; MHC-matched or MHC-mismatched). In this co-culture system, PBLs were not mitogen-activated in order to assess their responsiveness upon re-encountering with the same MSCs. Such response was assessed in terms of proliferative response of different lymphocyte subsets and production of IFNγ by T cells. To do this, PBLs were stained with carboxyfluorescein succinimidyl ester (CFSE) to evaluate their proliferation after 5 days of co-culture with MSCs. At this point, PBLs were collected and labelled with a panel of antibodies to analyze by flow cytometry the changes in the frequency and proliferation of the main lymphocyte subsets (T cells: cytotoxic, helper and regulatory, and B cells). At the same time, co-culture supernatants were collected to determine the secretion of IFNγ by ELISA, serving as an indicator of T cell activation (Figure 2B).

### 2.2 Animal selection by MHC-haplotyping

Twenty healthy horses aged between 2 to 12 years (12 geldings, 7 mares and 1 stallion), with no previous history of receiving MSCs or transfusions, were selected based on their MHC haplotypes as determined by microsatellite typing using a validated panel of 10 highly polymorphic intra-MHC regions, as previously described (12, 35). The methodology for DNA isolation, multiplex polymerase chain reaction (PCR), and fragment analysis was performed exactly as previously reported by our group (17).

Definitive haplotypes were established for animals homozygous or with direct relatives (parent-offspring pairs or half-sibling groups) and the remaining animals were assigned with provisional haplotypes based on previously known ones reported in bibliography (12, 14, 35, 36). Supplementary material S1 shows the microsatellite alleles of each haplotype identified in the different horses.

All the procedures involving animals were carried out under the Project License PI 15/16 approved by the in-house Advisory Ethics Committee for Animal Research from the University of Zaragoza. The care and use of animals were performed accordingly with the Spanish Policy for Animal Protection RD118/2021, which meets the European Union Directive 2010/63. All animals were kept on paddocks of the facilities of the Animal Research Service of the University of Zaragoza, with free access to water and fed *ad libitum* with grass hay.

### 2.3 Preparation of MSCs for *in vivo* administration: MSC-naïve, MSC-primed and MSC-chondro

Equine BM-MSCs were obtained and characterized, as previously described (23), as part of a previous study of our group (17). In brief, equine MSCs (*n* = 3) were isolated and expanded at 5,000 cells/cm^2^ in conventional MSCs medium consisting of low glucose Dulbecco’s Modified Eagle’s Medium (DMEM) supplemented with 10% fetal bovine serum (FBS), 2 mM L-glutamine, 0.1 mg/mL streptomycin and 100 U/mL penicillin (all form Sigma–Aldrich) at 37°C and 5% CO_2_.

Mesenchymal stem/stromal cells were used between passage two to four. Cells were detached using trypsin-EDTA 0.25% (Sigma–Aldrich), suspended at a concentration of 5 × 10^6^ MSCs/mL in phosphate buffered saline (PBS, Gibco, Thermo Fisher), and combined in a 1:1 ratio with a 3% alginate solution (ultrapure low-viscosity 67% guluronate, UPLVG; NovaMatrix, FMC Corporation). This resulted in a final concentration of 1.5% alginate in a total volume of 2 mL per scaffold as previously described (32), containing 5 × 10^6^ MSCs each scaffold. Scaffold polymerization was achieved as described earlier (37–39) by incubating them for 30 min with a solution of 102 mM CaCl_2_ (Sigma–Aldrich) at 37°C and 5% CO_2_. For MSC-naïve and MSC-primed, scaffolds were maintained in conventional MSC culture medium for 48 h. For MSC-primed scaffolds, 12 h before their *in vivo* placement, 5 ng/mL of TNFα and IFNγ (R&D Systems) were added to the conventional MSC media according to previous reports (22). For MSC-chondro, chondrogenesis was induced according to the methodology previously described (18, 37) using 10 ng/mL of TGFβ-3 (PeproTech) for 21 days.

To remove xenogeneic antigens from the FBS that might affect the immune response, 24 h prior to the *in vivo* placement of the scaffolds, all of them (MSC-naïve, MSC-primed, and MSC-chondro) were washed twice with PBS and fresh culture media was added containing 10% autologous serum from each recipient to replace the FBS (40). Right immediately before the intervention, scaffolds were washed again with PBS three times.

Scaffolds were placed along the neck tables, from cranial to caudal, following the previously described subcutaneous pocket technique (41). The surgical intervention was carried out on station under appropriate sedation (detomidine 0.01 mg/kg, Sedaquick, Fatro; and butorphanol 0.02 mg/kg, Torbugesic, Pfizer) and local anesthesia (lidocaine 5%, Braun). For the placement of each scaffold, a longitudinal incision of approximately 2 cm in length was performed, leaving about 10 cm between incisions. Subcutaneous tissue was dissected distally to the incision to create a subcutaneous pocket of approximately 2.5 × 2.5 cm, where each scaffold was placed. The incisions were closed in two layers (subcutaneous and skin) with 2/0 USP polyglyconate suture and surgical staples. This method of administration was chosen to facilitate the analysis of implant responses in a separate study. All horses were clinically monitored and received a single dose of flunixin meglumine IV (1.1 mg/kg, Niglumine, Calier) right before surgery and procaine penicillin IM (15 mg/kg once daily for 3 days, Procapen, Livisto) postoperatively.

### 2.4 Co-cultures of equine MSCs and lymphocytes: modified one-way MLR assays

One-way MLRs were conducted before and serially after each MSC administration for a total of 8 time-points (T0 to T7). The co-cultures were carried out following the methodology previously validated by our group (17).

#### 2.4.1 Preparation of MSC-naïve, MSC-primed and MSC-chondro for modified one-way MLR co-cultures

To conduct the co-cultures with fresh PBLs at each time-point, cryopreserved MSCs (*n* = 3) were thawed and cultured in standard conditions for 72 h to recover from freezing. Subsequently, MSCs were detached with 0.25% trypsin-EDTA and seeded into a 24-well plate at 20,000 MSCs per well. For MSC-naïve and MSC-primed, plating was done 24 h prior to starting the co-cultures to allow their attachment to the well. For MSC-primed, MSCs were exposed for 12 h to 5 ng/mL of equine recombinant TNFα plus 5 ng/mL of equine recombinant IFNγ (23). For MSC-chondro, plating was performed with the same number of cells but 14 days prior to starting the co-cultures to induce differentiation with the StemPro^™^ Chondrogenesis Differentiation Kit (Thermo Fisher) and using the micro-mass system (42). Each MSC type was prepared to run in duplicate each co-culture with corresponding recipients’ PBLs. Prior to adding the PBLs, wells were washed with PBS to remove components of each specific media.

#### 2.4.2 Isolation of PBLs and CFSE labelling

At the designated time-points, blood was collected aseptically from all recipients via jugular venipuncture and PBLs were isolated using the carbonyl iron granulocyte depletion method, followed by density gradient centrifugation with Lymphoprep^™^ (Fisher Scientific) as previously used by our group (17). This isolation technique has been reported to provide an enriched lymphocyte population of 95–99% (40).

Subsequently, PBLs were labelled with 2.5 μM CFSE (Sigma–Aldrich) in order to evaluate lymphocyte proliferation by assessing CFSE dilution using flow cytometry (13, 20). After the staining procedure, PBLs were counted in a hemocytometer chamber using Trypan Blue 0.4% and adjusted to 10 × 10^6^ live cells/mL in PBL medium (consisting of RPMI 1640 medium supplemented with 10% FBS, 0.1 mM 2-mercaptoethanol, 100 U/mL penicillin, and 100 μg/mL streptomycin, all from Sigma–Aldrich).

#### 2.4.3 Modified one-way MLR

One million of responder PBLs were added per well, each one containing 20,000 stimulator MSCs of the corresponding type as described above, to obtain a MSC:PBL ratio of 1:50 (13, 33). Appropriate PBL controls were established in duplicate for each recipient, using 500,000 PBLs per well: unlabeled PBLs to account for background signal, CFSE-labelled PBLs alone to set the non-proliferating gate, and CFSE-labelled PBLs stimulated with 10 μg/mL of the mitogen phytohemagglutinin isoform P (PHA, Sigma–Aldrich) as internal positive control to corroborate that PBLs were able to proliferate (17, 43). In addition, classic MLRs were established for each recipient as control. Briefly, MHC-matched and mismatched PBLs were used as stimulators by treating them with 50 μg/mL mitomycin C (Sigma–Aldrich) (37°C 30 min incubation followed by 2 washes with PBS) to inhibit proliferation (14, 44). Stimulator PBLs were plated at 500,000 PBLs/well in 96-well plates immediately before the addition of 500,000 CFSE-stained responder PBLs (ratio 1:1) to create parallel MHC-matched and mismatched classic MLRs for all the PBLs (17). All the co-cultures were carried out with freshly isolated PBLs and were maintained for 5 days without changing media.

### 2.5 Analysis of lymphocyte subpopulations frequency and proliferation

After 5 days in modified one-way MLR co-cultures, the PBLs were collected from the 24-well plates, centrifuged at 310× g for 5 min, resuspended in PBS, and split for the two flow multi-color panels. The supernatants were collected and centrifuged at 500× g for 15 min to remove any contaminating cell and were immediately frozen at −20°C for further ELISA analysis, as will be detailed below. Control PBLs (alone) and their supernatants were processed in the same way.

Two multi-color panels of markers were previously designed by our group to allow assessment of different lymphocyte subpopulations, along with the proliferation (CFSE dilution) (17). In panel 1, anti-CD3 (primary rat anti-human, 1:100, MCA1477, Bio-Rad; secondary mouse anti-rat, 1:100, 12-4812-82, Invitrogen), anti-Pan Ig (primary mouse anti-horse, 1:100, MCA1899, Bio-Rad; secondary goat anti-mouse, 1:200, A-21036, Invitrogen) and anti-CD21 (mouse anti-human, 1:40, 561357, BD Pharmigen) were used to assess the global T and B cell populations. In panel 2, anti-CD8 (mouse anti-horse, 1:5, MCA2385, Bio-Rad), anti-CD4 (primary mouse anti-horse, 1:200, MCA1078, Bio-Rad; secondary rat anti-mouse, 1:200, 406619, BioLegend) and anti-CD25 (primary goat anti-human, 1:50, AF-223, R&D Systems, secondary donkey anti-goat, 1:400, 705-605-003, Jackson Immuno Research) antibodies were used to assess cytotoxic, helper and regulatory T cells, respectively.

All the primary antibodies were selected based on previous reports and previously checked to correctly label equine cells. Primary antibodies were used directly conjugated with fluorochromes or in combination with appropriate secondary antibodies (34, 45, 46). The methodology for staining the equine PBLs with these antibodies has been described in detail in a previous publication of our group that can be openly accessed, and was exactly the same than in this study (17).

All samples were analyzed in a Gallios flow cytometer (Beckman Coulter, Madrid, Spain), acquiring a minimum of 10,000 events per sample. Flow cytometry data was analyzed with FCS Express 7 Flow software (*De Novo* Software, Pasadena, CA, United States). Unstained PBLs and secondary controls (cells stained with secondary antibodies alone) were used to assess fluorescence background and to establish gates for each marker. Compensation controls were performed using single color stains, while fluorescence minus one (FMO) controls were used to determine the fluorescence spread from other channels. Viability staining was performed with Ghost dye Violet 450 (Tonbo Biosciences, Bio-Rad) for all PBLs.

The gating strategy was the same than in a previous report from our group (17). Briefly, the lymphocyte population was initially gated in the forward and side scatter (FSC × SSC) plot, with doublets being excluded from analysis, followed by exclusion of dead cells that incorporated the viability stain. In the first panel, live cells were further gated as T cells (CD3^+^) or B cells (CD3^−^/Pan Ig^+^/CD21^+^) (47). In panel 2, live cells were gated to differentiate between cytotoxic T cells (CD8^+^/CD4^−^) and helper T cells (CD4^+^/CD8^−^). Furthermore, the subpopulation of regulatory T cells (Treg) was gated from the CD4^+^ cell population based on their high expression of CD25. The subpopulation of CD4^+^ CD25^high^ is referred as Treg cells in this study for clarity; however, it should be noted that other subpopulations may be present in this gate.

Frequency (%) of each lymphocyte subset over the total lymphocyte population was recorded for all recipients at all time-points. To evaluate the proliferation of PBLs in each specific lymphocyte subset, CFSE dilution was analyzed in cells gated as aforementioned. Autofluorescence (background) was determined using unstimulated and unstained PBLs. Unstimulated and CFSE-labeled PBLs were used to establish the non-proliferating population, considering cells to the left (lower fluorescence intensity) as the proliferating population.

To account for inter-individual variability, the percentage and proliferation of each lymphocyte subpopulation after exposure to MSCs were normalized to the values from classic MLR-matched controls (assigned as value 1). Unspecific proliferation was not observed in the MLR-M controls at any time-point.

### 2.6 Interferon gamma secretion assay

Supernatants collected from the one-way MLR assays and corresponding controls were used to evaluate IFNγ production by using a commercially available ELISA kit (Equine IFN-gamma DuoSet ELISA, R&D Systems, REF: DY1586), as previously reported (17, 44, 48).

The supernatants from the classic MLRs with MHC-matched or mismatched PBLs as stimulators were used as the negative and positive control, respectively. All supernatants were diluted 1:1 in reagent diluent. All the procedures were performed as per manufacturer’s instructions and concentrations determined using a standard curve, including a blank.

The standard curve was set from 62.5 pg/mL to 8,000 pg/mL of IFNγ. All the samples and points of the standard curve were run in triplicate. All the colorimetric assays were analyzed on a microplate reader (Biotek Synergy HT, Winooski, VA, United States) and read immediately at 450 nm with wavelength correction set to 540 nm. The duplicate readings for each standard, control, and sample were averaged, and the average zero standard optical density was extracted. The standard curve was created generating a four-parameter logistic curve-fit and the concentrations extrapolated were multiplied by the dilution factor.

### 2.7 Statistical analysis

Statistical analysis was performed with IBM SPSS Statistics version 26 statistical package. Analytical statistical tests were used to test for differences in each lymphocyte subpopulation and in IFNγ secretion as the dependent variables of the study.

The independent variables were “MHC group” (two categories: MHC-matched and MHC-mismatched), “MSC type” (three categories: MSC-naïve, MSC-primed and MSC-chondro) and “time” (eight categories: T0, T1, T2, T3, T4, T5, T6, T7). Each dependent variable was analyzed individually and each of the independent variables was analyzed as a factor, to study the differences as follows: differences between MSC types at each time within each MHC group of recipients, differences between MHC groups of recipients for each MSC type at each time, differences over time within each MHC group of recipients for each MSCs type.

The existence of outlier samples was evaluated with the Grubbs test (alpha = 0.05) and the results were analyzed by Shapiro–Wilk test to assess normality of data. Levene’s test was used to test the equality of variances. When data followed a normal distribution and had homogeneous variances, the parametric test ANOVA was used, followed by Bonferroni comparisons test as a *post hoc*. In normally distributed data with unequal variances, Welch’s *t*-test was used. In non-normal data and variables with more than three groups, Kruskal–Wallis or Friedman tests followed by Dunn’s test were used as *post hoc*, for independent (differences between MSC types) or related (differences over time) samples, respectively. The Mann–Whitney *U* test was used for comparisons between two groups (differences between MHC groups). The significance level was set at *p* < 0.05 for all analyses. GraphPad Prism 9.2 was used for graphical representation (San Diego, CA, United States).

## 3 Results

Proliferative response of the different lymphocyte subsets and IFNγ secretion are presented in this section. Data regarding changes in the frequency (%) of lymphocyte subsets are presented in Supplementary material S2. These changes were mild compared to the proliferative response, highlighting the value of assessing the latter as a closer reflection of the immune response. To prevent any confusion and to lighten the volume of data here included, authors chose to present the proliferative response in the main text and the population frequencies as Supplementary material.

### 3.1 Proliferative response of lymphocyte subpopulations after *in vitro* re-exposure to the MSCs administered *in vivo* (modified one-way MLR assay)

This study evaluated the effect of two allogeneic administrations of MSCs under different conditions (naïve, primed and chondro) and under different MHC combinations (matched and mismatched) on the response of circulating PBLs. To this end, the proliferation of different lymphocyte populations from recipient horses was measured at different time-points using an *in vitro* co-culture system with the same MSCs administered *in vivo*.

#### 3.1.1 CD3^+^ T lymphocytes: proliferative response increases after the second administration and particularly in MHC-mismatched recipients

**Figure 3:**
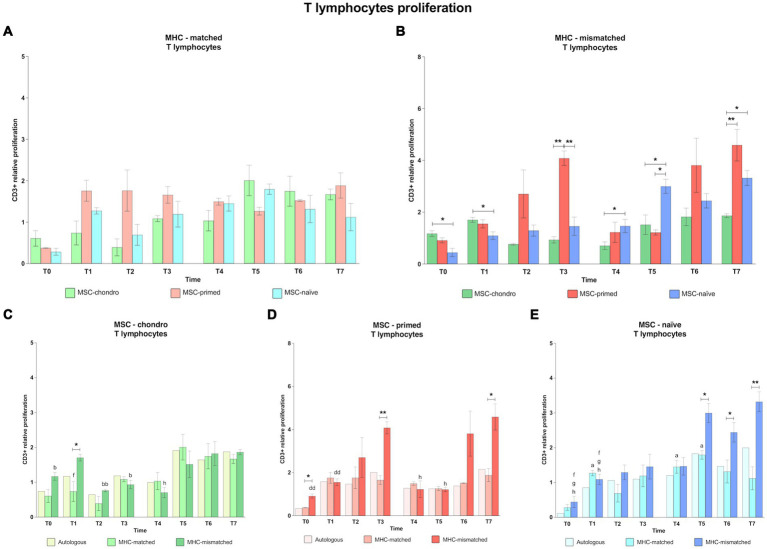
Proliferation of CD3^+^ T lymphocytes. Mean ± SEM of the relative proliferation of CD3^+^ T lymphocytes in the immunogenicity assays (modified one-way mixed lymphocyte reaction) in MHC-matched **(A)** and MHC-mismatched **(B)** recipients following the administration of MSC-chondro (green bars), MSC-primed (orange bars) and MSC-naïve (blue bars). Changes along time of non-activated PBLs from autologous, MHC-matched and MHC-mismatched recipients exposed *in vitro* to MSC-chondro **(C)**, MSC-primed **(D)** and MSC-naïve **(E)**. Proliferation of each PBL donor is normalized over the proliferation observed in the negative control (MLR M−, matched MLR) consisting of responder PBLs from the same donor exposed to MHC-matched stimulator PBLs (value 1), to account for inter-individual variability. Significant differences between cell-type and groups at one time-point are represented by a squared line with an asterisk (^*^p < 0.05 and ^**^p < 0.01). Significant differences between time-points are represented by lower case letter: T0, ^a^; T1, ^b^; T3, ^d^; T5, ^f^; T6, ^g^ and T7, ^h^ (^a^, ^b^, ^f^, ^g^, ^h^, *p* < 0.05; ^bb^, ^dd^, *p* < 0.01).

In general, CD3^+^ proliferation tended to be higher after the second administration for all types of MSCs and MHC combinations, even in the autologous control groups (Figure 3). The different MSC types (naïve, primed and chondro) produced similar CD3^+^ proliferation in the MHC-matched recipients, but MSC-primed induced more response after the first administration and MSC-chondro after the second one (Figure 3A). On the other hand, MHC-mismatched administration promoted a higher T cell response (Figure 3B): for the first administration, MSC-chondro initially induced a significantly increased proliferation compared to MSC-naïve (T0 and T1, *p* < 0.05), while at later time-points MSC-primed induced more T cell proliferation than both MSC-chondro and MSC-naïve (T3, *p* < 0.01). However, in the second administration of MHC-mismatched cells, MSC-naïve promoted higher T cell proliferation (T4 and T5 over MSC-chondro; T5 over MSC-primed, *p* < 0.05). Nevertheless, at the last time-point analyzed, T cell response was higher against MSC-primed (T7 over MSC-chondro, *p* < 0.01) followed by MSC-naïve (T7 over MSC-chondro, *p* < 0.05).

When considering separately the response elicited by each MSC type, MSC-chondro (Figure 3C) induced a significant increase in CD3^+^ proliferation after the second administration, even in MHC-matched recipients (T5 over T1, *p* < 0.05). Moreover, when the recipients were MHC-mismatched, this response was already observed after the first administration (T1 over: T0, *p* < 0.05; T2, *p* < 0.01; T3, *p* < 0.05), being significantly higher than in the MHC-matched group (T1, *p* < 0.05), and increased until the last time-point evaluated (T7 over T4, *p* < 0.05).

For MSC-primed (Figure 3D), MHC-mismatched recipients also showed a higher proliferative response. However, compared to MSC-chondro, the response took place later and its degree was similar between the first and the second administration (T3 over T0 and T1, *p* < 0.01; T7 over T4 and T5, *p* < 0.05). MHC-mismatched recipients presented a higher T cell response already at the baseline (T0, *p* < 0.05) but, afterwards, the response was similar between MHC-matched and mismatched groups until reaching 6 weeks after each administration, when CD3^+^ proliferation was higher in the MHC-mismatched group (T3, *p* < 0.01; T7, *p* < 0.05).

For MSC-naïve (Figure 3E), the first administration induced a proliferative response over the baseline (T0), but this response was similar between MHC-matched and mismatched groups. MHC-matched co-cultures showed a significant increase over the baseline at different moments (T1, T4 and T5, *p* < 0.05). In MHC-mismatched recipients, the second administration of MSC-naïve produced an increase in CD3^+^ T cell proliferation (T5, T6 and T7 over T0 and T1, *p* < 0.05) that was also significant over the MHC-matched group (T5 and T6, *p* < 0.05; T7, *p* < 0.01).

#### 3.1.2 CD8^+^ cytotoxic and CD4^+^ helper T lymphocytes: MSC-primed and MHC-mismatching induce higher helper and cytotoxic responses

**Figure 4:**
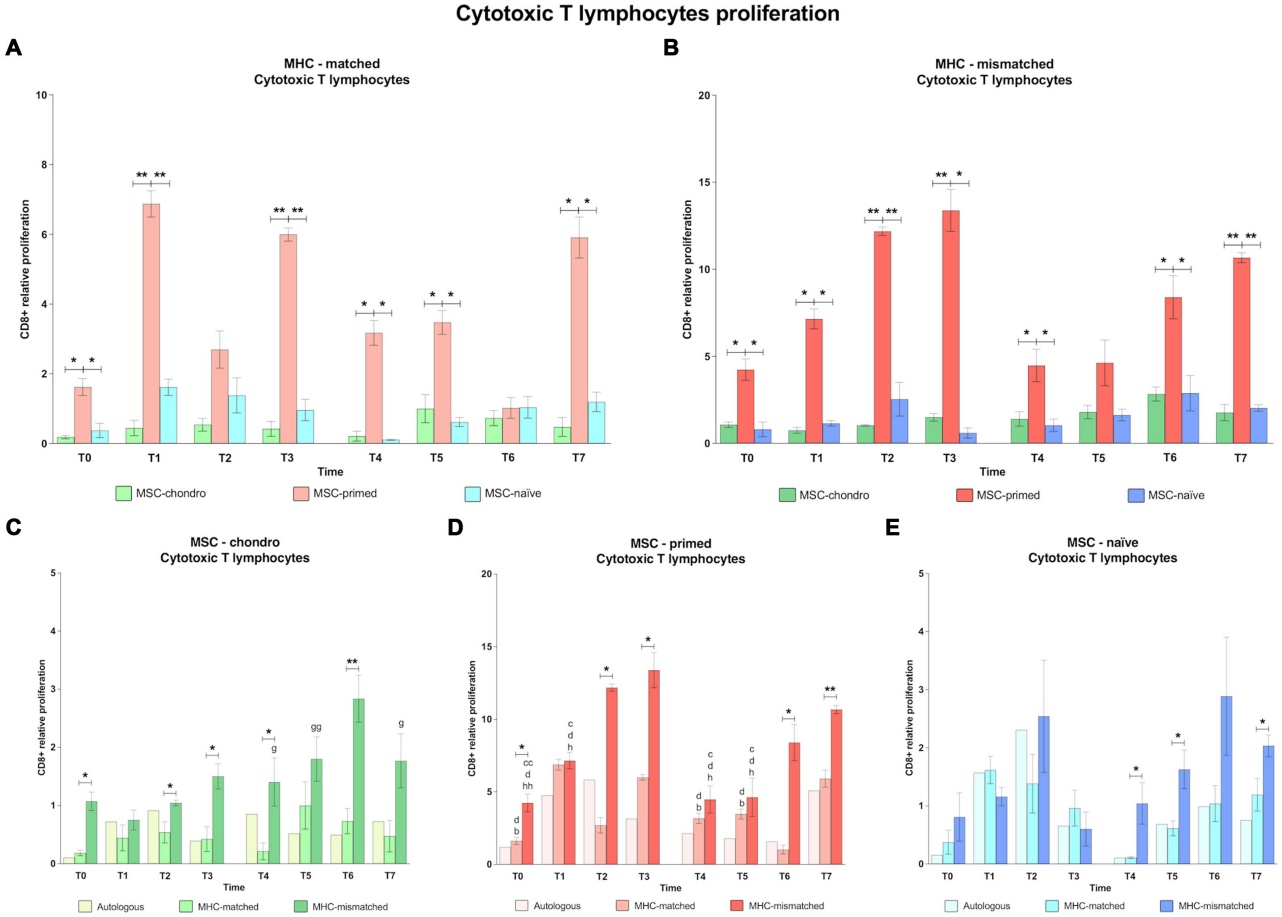
Proliferation of CD8^+^ cytotoxic T cells. Mean ± SEM of the relative proliferation of CD8^+^ cytotoxic T cells in the immunogenicity assays (modified one-way mixed lymphocyte reaction) in MHC-matched **(A)** and MHC-mismatched **(B)** recipients following the administration of MSC-chondro (green bars), MSC-primed (orange bars) and MSC-naïve (blue bars). Changes along time of non-activated PBLs from autologous, MHC-matched and MHC-mismatched recipients exposed *in vitro* to MSC-chondro **(C)**, MSC-primed **(D)** and MSC-naïve **(E)**. Proliferation of each PBL donor is normalized over the proliferation observed in the negative control (MLR M−, matched MLR) consisting of responder PBLs from the same donor exposed to MHC-matched stimulator PBLs (value 1), to account for inter-individual variability. Significant differences between cell-type and groups at one time-point are represented by a squared line with an asterisk (^*^p < 0.05 and ^**^p < 0.01). Significant differences between time-points are represented by lower case letter: T1, ^b^; T2, ^c^; T3, ^d^; T6, ^g^ and T7, ^h^ (^b^, ^c^, ^d^, ^g^, ^h^, *p* < 0.05; ^cc^, ^gg^, ^hh^, *p* < 0.01).

**Figure 5:**
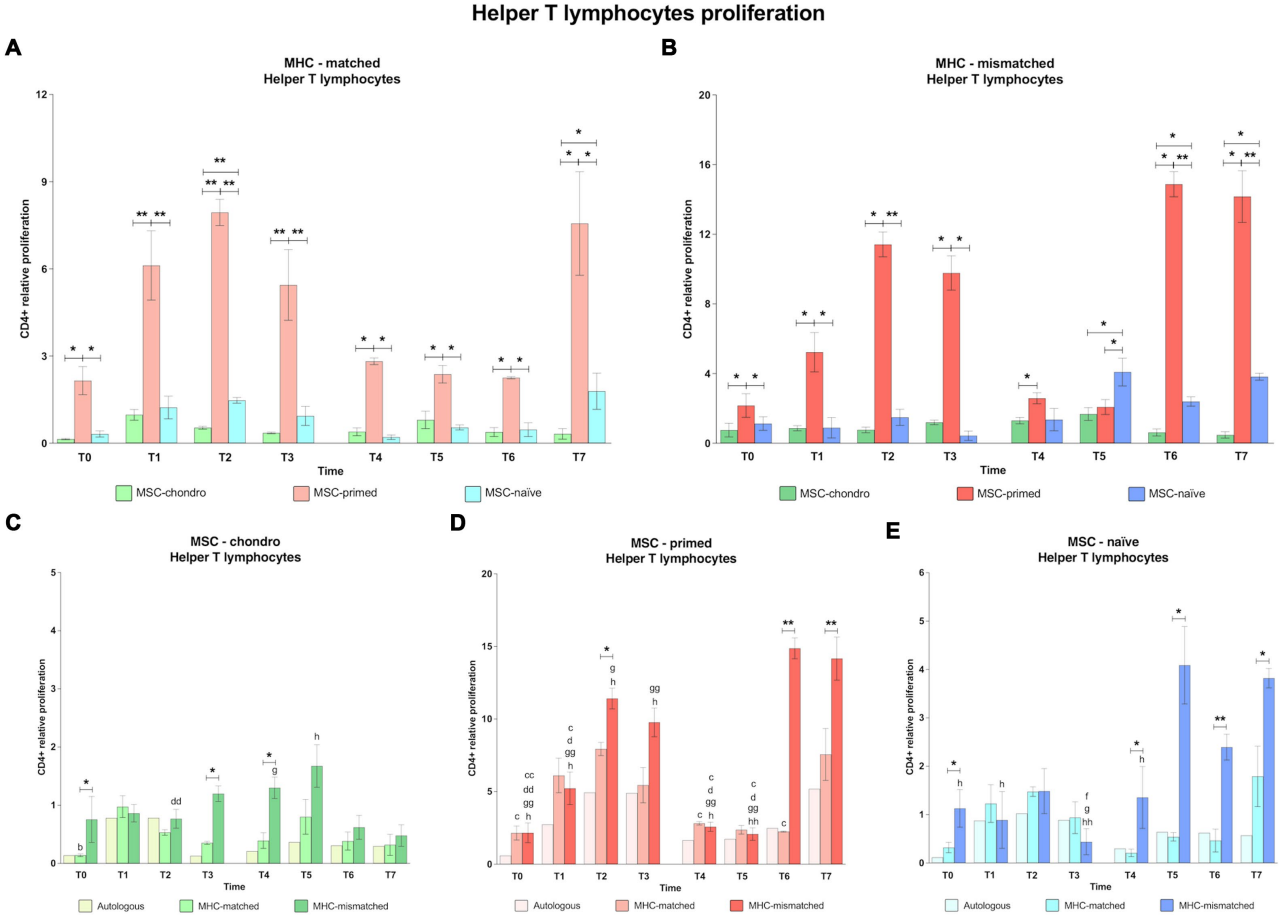
Proliferation of CD4^+^ helper T cells. Mean ± SEM of the relative proliferation of CD4^+^ helper T cells in the immunogenicity assays (modified one-way mixed lymphocyte reaction) in MHC-matched **(A)** and MHC-mismatched **(B)** recipients following the administration of MSC-chondro (green bars), MSC-primed (orange bars) and MSC-naïve (blue bars). Changes along time of non-activated PBLs from autologous, MHC-matched and MHC-mismatched recipients exposed *in vitro* to MSC-chondro **(C)**, MSC-primed **(D)** and MSC-naïve **(E)**. Proliferation of each PBL donor is normalized over the proliferation observed in the negative control (MLR M−, matched MLR) consisting of responder PBLs from the same donor exposed to MHC-matched stimulator PBLs (value 1), to account for inter-individual variability. Significant differences between cell-type and groups at one time-point are represented by a squared line with an asterisk (^*^p < 0.05 and ^**^p < 0.01). Significant differences between time-points are represented by lower case letter: T1, ^b^; T2, ^c^; T3, ^d^; T5, ^f^; T6, ^g^ and T7, ^h^ (^b^, ^c^, ^d^, ^f^, ^g^, ^h^, *p* < 0.05; ^cc^, ^dd^, ^gg^, ^hh^, *p* < 0.01).

Along all the time-points analyzed, MSC-primed clearly promoted the highest CD4^+^ T and CD8^+^ T cell response in both MHC-matched and mismatched recipients, followed by MSC-chondro and MSC-naïve (Figures 4, 5). Importantly, MHC-matched recipients presented lower proliferation of helper and cytotoxic T cells against all the three types of MSCs. Furthermore, the proliferative response after the second *in vivo* administration of MHC-mismatched MSCs was more marked for MSC-chondro compared to the first administration, while it was attenuated for MSC-primed.

In the MHC-matched group (Figures 4A, 5A), MSC-primed induced significantly higher proliferation of both CD8^+^ and CD4^+^ T cells compared to both MSC-naïve and MSC-chondro at all time-points (T0, *p* < 0.05; T1, *p* < 0.01; T2 only CD4^+^, *p* < 0.01; T3, *p* < 0.01; T4, T5, T6 (only CD4^+^) and T7, *p* < 0.05). Higher CD4^+^ proliferation was also observed for MSC-naïve over MSC-chondro (T2, *p* < 0.01; T7, *p* < 0.05) (Figure 5A).

Regarding the MHC-mismatched group (Figures 4B, 5B), CD8^+^ T and CD4^+^ T cell proliferation followed a pattern similar to the MHC-matched group, in which MSC-primed also induced a significantly higher response over MSC-chondro (both CD8^+^ and CD4^+^: T0, T1, T4 and T6, *p* < 0.05; CD8^+^: T2, T3 and T7, *p* < 0.01; CD4^+^: T2, T3, and T7, *p* < 0.05) and over MSC-naïve (both CD8^+^ and CD4^+^: T0, T1 and T3, *p* < 0.05; T2 and T7, *p* < 0.01; CD8^+^: T4 and T6, *p* < 0.05; CD4^+^: T6, *p* < 0.01). There was an exemption at T5 in which the highest proliferation of CD4^+^ T cells was promoted by MSC-naïve (*p* < 0.05 over both MSC-primed and MSC-chondro). MSC-naïve also induced higher helper response than MSC-chondro at later time-points (T6 and T7, *p* < 0.05) (Figure 5B). On the other hand, no significant differences in the proliferation of CD8^+^ T cells were observed between MSC-chondro and MSC-naïve (Figure 4B).

In the analysis of each MSC type separately, we observed that MSC-chondro co-cultured with MHC-matched lymphocytes only induced a significant increase in helper T cell proliferation at 1 week after the first administration (T1 over T0, *p* < 0.05), but this response did not exceed the MLR-matched values set as control (value 1) (Figure 5C). In contrast, administration of MSC-chondro to MHC-mismatched recipients induced greater changes in CD8^+^ and CD4^+^ proliferation over time (Figures 4C, 5C). Specifically, CD4^+^ proliferation progressively increased (T3 over T2, *p* < 0.01), peaked at T5 (1 week after second administration), and decreased afterwards (T6 over T4; T7 over T5, *p* < 0.05) (Figure 5C). CD8^+^ proliferation in MSC-chondro co-cultures also increased progressively but peaked later (T6 over: T4, *p* < 0.05; T5, *p* < 0.01; T7, *p* < 0.05) (Figure 4C). Higher cytotoxic and helper T cell proliferation was observed in MHC-mismatched co-cultures compared to the MHC-matched group (both CD8^+^ and CD4^+^: T0, T3, T4, *p* < 0.05; CD8^+^: T2, T6, *p* < 0.01) (Figures 4C, 5C).

For MSC-primed, MHC-mismatched recipients overall showed higher cytotoxic and helper responses compared to the MHC-matched group (both CD8^+^ and CD4^+^: T2, *p* < 0.05; T6, *p* < 0.05 CD8^+^ and *p* < 0.01 CD4^+^; T7, *p* < 0.01; CD8^+^: T0 and T3, *p* < 0.05). Interestingly, the proliferative response was more marked for cytotoxic T cells after the first administration, and for helper T cells after the second one. In both cases, the proliferative response tended to increase towards the later time-points analyzed. Actually, the proliferation of CD4^+^ T cells was very mild at 1 week after each administration of MSC-primed in both MHC-matched and mismatched recipients, while after 3 weeks and 6 weeks after each administration the MHC-mismatched group markedly increased the T helper response (first administration: T2 and T3 over: T0, *p* < 0.01; T1, T4 and T5, *p* < 0.05; second administration: T6 over: T0, T1, T3, T4, T5, *p* < 0.01; T2, *p* < 0.05; T7 over: T0, T1, T2, T3, T4, *p* < 0.05; T5, *p* < 0.01) (Figure 5D). MHC-mismatched co-cultures also induced a significant proliferation increase of CD8^+^ (over T0: T2 *p* < 0.01, T3 *p* < 0.5 and T7 *p* < 0.01; T2, T3 and T7 over: T1, T4 and T5, *p* < 0.05) (Figure 4D). Nonetheless, MHC-matched recipients also presented an increased response of cytotoxic T cells (T1 and T3 over T0, T4, and T5, *p* < 0.05) (Figure 4D) and of helper T cells (T2 over T0, T4 and T6, *p* < 0.05) (Figure 5D).

MSC-naïve administration generally induced a similar CD4^+^ proliferation after both the first and the second administration in the MHC-matched group. However, in the MHC-mismatched group, the second administration produced higher CD4^+^ proliferation compared to the first exposure at all the time-points (over T3: T5 and T6, *p* < 0.05; T7, *p* < 0.01) (Figure 5E), especially at the last one (T7 over: T0, T1 and T4, *p* < 0.05) (Figure 5E), while the response of cytotoxic T cells was less marked (Figure 4E). There were no significant differences between MHC-matched and MHC-mismatched recipients for the first administration, even though they were observed at the baseline (T0, *p* < 0.05 for CD4^+^ cells) (Figure 5E). This was also observed prior to the second administration (T4, *p* < 0.05 for both CD8^+^ and CD4^+^ cells) (Figures 4E, 5E) but, in contrast to the first administration, MHC-mismatched recipients showed a progressive increase in the proliferative response of both helper and cytotoxic T cells after the second administration (T5 and T7, *p* < 0.05 for both CD8^+^ and CD4^+^; T6, *p* < 0.01 only CD4^+^) (Figures 4E, 5E).

#### 3.1.3 CD4^+^ CD25^high^ regulatory T cells: MSC-primed have a greater capacity to induce Treg, particularly in the MHC-mismatched condition, whereas MSC-chondro barely modifies this population

The effect of the MSC type after the first administration of MHC-matched cells could not be analyzed on Treg cells at T1 and T2. This was due to disruptions in the supply chain of the anti-CD25 antibody, for reasons external to the authors’ control. Since all the co-cultures were performed with fresh PBLs at the different time-points, it was not possible to conduct these analyses later.

**Figure 6:**
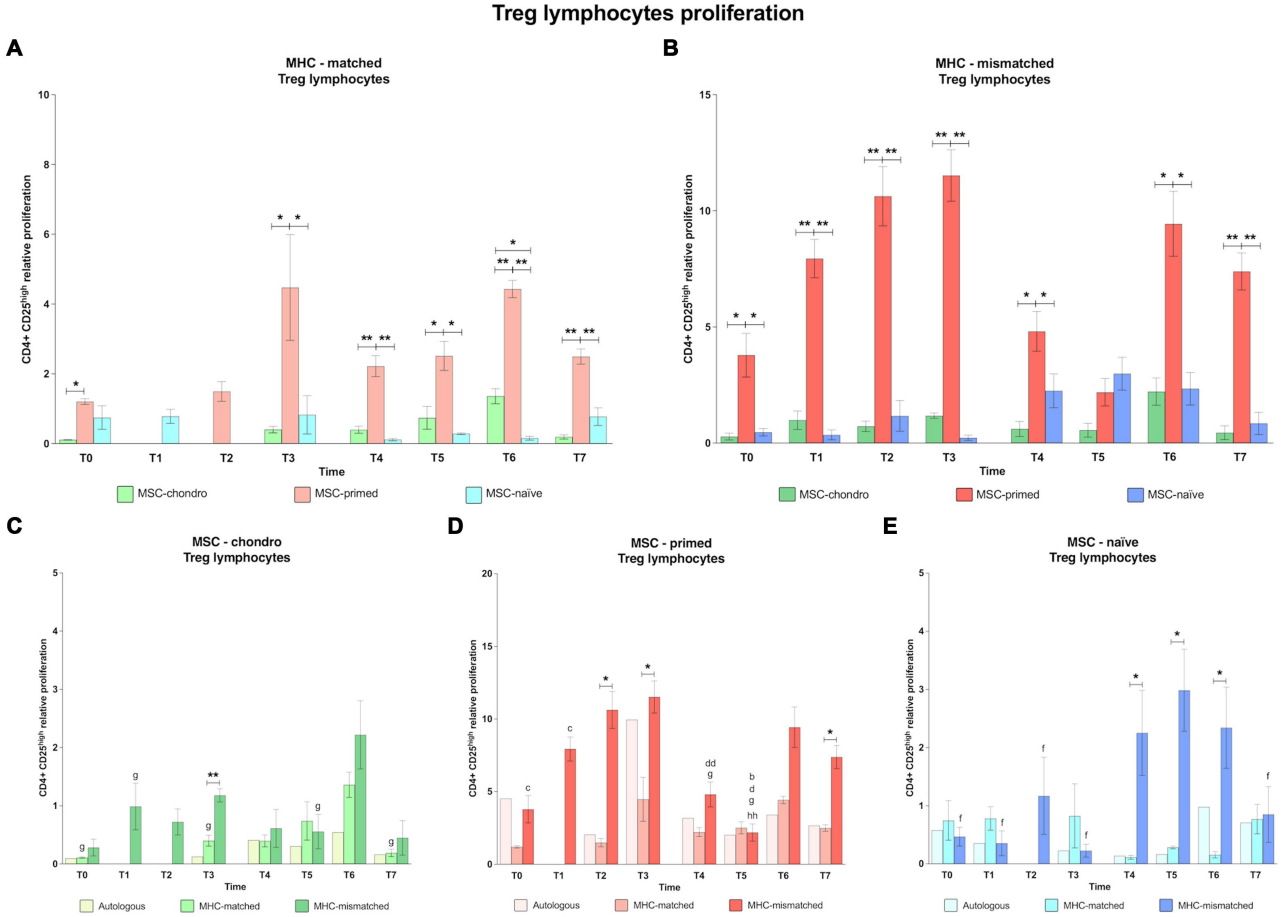
Proliferation of CD4^+^ CD25^high^ regulatory T cells. Mean ± SEM of the relative proliferation of CD4^+^ CD25^high^ regulatory T cells in the immunogenicity assays (modified one-way mixed lymphocyte reaction) in MHC-matched **(A)** and MHC-mismatched **(B)** recipients following the administration of MSC-chondro (green bars), MSC-primed (orange bars) and MSC-naïve (blue bars). Changes along time of non-activated PBLs from autologous, MHC-matched and MHC-mismatched recipients exposed *in vitro* to MSC-chondro **(C)**, MSC-primed **(D)** and MSC-naïve **(E)**. Proliferation of each PBL donor is normalized over the proliferation observed in the negative control (MLR M−, matched MLR) consisting of responder PBLs from the same donor exposed to MHC-matched stimulator PBLs (value 1), to account for inter-individual variability. Significant differences between cell-type and groups at one time-point are represented by a squared line with an asterisk (^*^p < 0.05 and ^**^p < 0.01). Significant differences between time-points are represented by lower case letter: T1, ^b^; T2, ^c^; T3, ^d^; T5, ^f^; T6, ^g^ and T7, ^h^ (^b^, ^c^, ^d^, ^f^, ^g^, *p* < 0.05; ^hh^, *p* < 0.01).

In general, MSC-primed tended to markedly increase Treg proliferation, whereas MSC-chondro showed minimal activation of Treg, regardless of the MHC combination (Figures 6A,B). Already at the baseline (T0), MSC-primed activated Treg in both MHC-matched (over MSC-chondro, *p* < 0.05; Figure 6A) and MHC-mismatched scenarios (over both MSC-chondro and MSC-naïve, *p* < 0.05; Figure 6B). After both administrations, MSC-primed induced a significantly higher proliferation of Treg over both MSC-naïve and MSC-chondro at almost all the time-points in both MHC-matched (T3, *p* < 0.05, T4 *p <* 0.01, T5 *p* < 0.05, T6 and T7, *p* < 0.01; Figure 6A) and MHC-mismatched recipients (T1, T2 and T3, *p* < 0.01; T4 and T6, *p* < 0.05; T7, *p* < 0.05; Figure 6B). Of note, the Treg response in MHC-matched recipients of MSC-primed was higher after the second administration (Figure 6A), while in MHC-mismatched recipients it was higher after the first one (Figure 6B). MSC-chondro and MSC-naïve showed similarly mild activation of Treg, and significant differences between them were only punctually observed (T6 in MHC-matched recipients, *p* < 0.05; Figure 6A).

When analyzing each MSC type, we found that MSC-chondro (Figure 6C) tended to reduce Treg proliferation over time, compared to the MLR-matched control (value 1). An increase over this control was only observed at T6 in both MHC-matched and MHC-mismatched groups. This increase at T6 was also statistically significant over other time-points (MHC-matched co-cultures: T0, T3 and T7, *p* < 0.05; MHC-mismatched co-cultures: T1 and T5, *p* < 0.05). Overall, higher Treg proliferation was induced in MHC-mismatched compared to MHC-matched recipients along the time, but significant differences were only punctually found (T3, *p* < 0.01).

Regarding MSC-primed (Figure 6D), MHC-mismatched recipients overall presented a higher Treg response, which increased more markedly from 3 weeks onwards after each administration (T2 over: T0 and T1, *p* < 0.05; T6 over: T4 and T5, *p* < 0.05). At 6 weeks after the first administration (T3), the highest Treg induction was observed in MHC-mismatched recipients, which subsequently decreased (T4, *p* < 0.01; T5, *p* < 0.05) and was the lowest at T5 (over T1, *p* < 0.05; over T7, *p* < 0.01). On the other hand, in the MHC-matched group, Treg proliferation rates remained similar along the time and showed lower values for than MHC-mismatched recipients (T2, T3 and T7 *p* < 0.05).

In MSC-naïve recipients (Figure 6E), similar Treg response was observed after the first administration of either MHC-matched or MHC-mismatched cells. However, a Treg increase was observed in MHC-mismatched recipients after the second administration (T5 over T0, T1, T2, T3 and T7, *p* < 0.05). This response was significantly higher than in the MHC-matched group (T4, T5, and T6, *p* < 0.05).

#### 3.1.4 CD3^−^ Pan-Ig^+^ CD21^+^ B cells: MSC-chondro and MSC-naïve promote B cell activation, whereas MSC-primed have a limited stimulatory effect on B cells

**Figure 7:**
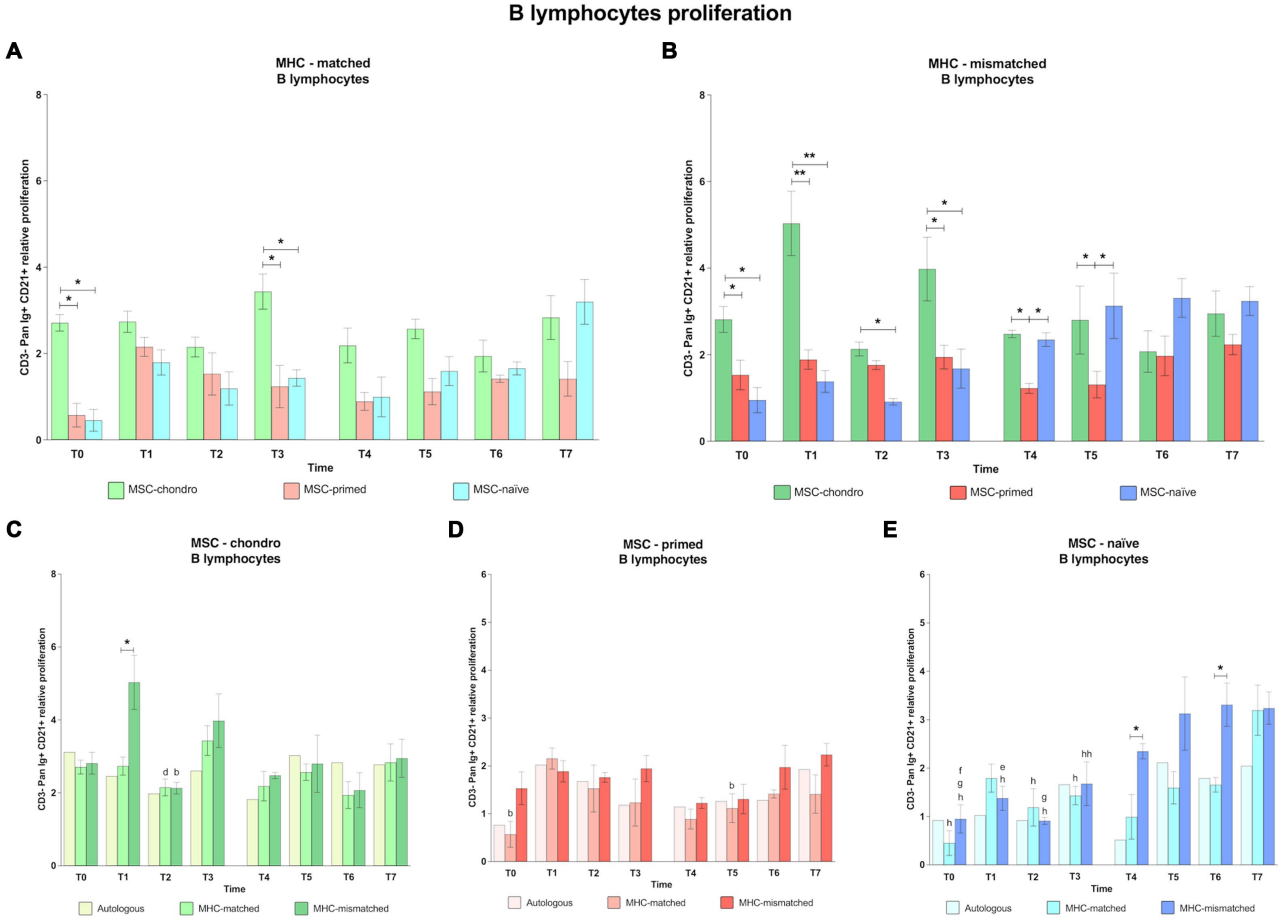
Proliferation of CD3^−^-Pan-Ig^+^ CD21^+^ B cells. Mean ± SEM of the relative proliferation of CD3^−^-Pan-Ig^+^ CD21^+^ B cells in the immunogenicity assays (modified one-way mixed lymphocyte reaction) in MHC-matched **(A)** and MHC-mismatched **(B)** recipients following the administration of MSC-chondro (green bars), MSC-primed (orange bars) and MSC-naïve (blue bars). Changes along time of non-activated PBLs from autologous, MHC-matched and MHC-mismatched recipients exposed *in vitro* to MSC-chondro **(C)**, MSC-primed **(D)** and MSC-naïve **(E)**. Proliferation of each PBL donor is normalized over the proliferation observed in the negative control (MLR M−, matched MLR) consisting of responder PBLs from the same donor exposed to MHC-matched stimulator PBLs (value 1), to account for inter-individual variability. Significant differences between cell-type and groups at one time-point are represented by a squared line with an asterisk (^*^p < 0.05 and ^**^p < 0.01). Significant differences between time-points are represented by lower case letter: T1, ^b^; T3, ^d^; T4, ^e^; T5, ^f^; T6, ^g^ and T7, ^h^ (^b^, ^d^, ^e^, ^f^, ^g^, ^h^, *p* < 0.05; ^hh^, *p* < 0.01).

MSC-chondro promoted in general the highest proliferation of B cells in both MHC-matched and MHC-mismatched recipients. The proliferation of B cells induced by either MSC-naïve or MSC-primed after the first administration was similar along time-points and between MHC-matched and MHC-mismatched groups. However, following the second administration, both MSC-chondro and MSC-naïve promoted a more marked response of B cells, whereas MSC-primed displayed the lowest B cell proliferation in both MHC-matched and MHC-mismatched groups (Figure 7).

When comparing the B cell response among the different types of MSCs, MSC-chondro induced a significantly higher response than both MSC-primed and MSC-naïve in most cases (both MHC-matched and MHC-mismatched groups: T0 and T3, *p* < 0.05; MHC-mismatched group: T1, *p* < 0.01, T2, *p* < 0.05 only over MSC-naïve) (Figures 7A,B). Similarly to MSC-chondro, MSC-naïve also increased B cell proliferation in the second administration, particularly in the MHC-mismatched group (T4 and T5 over MSC-primed, *p* < 0.05) (Figure 7B).

When analyzing each type of MSCs, MSC-chondro (Figure 7C) promoted a pattern of B cell activation similar between autologous, MHC-matched and MHC-mismatched recipients in most cases and with few exceptions. Such exceptions included a stronger reaction in MHC-mismatched recipients soon after the first administration of MSC-chondro, compared to the MHC-matched group (T1, *p* < 0.05). This B cell proliferation decreased in the next time-point (T2, *p* < 0.05) and increased again in the next one (T3 over T2 in MHC-matched group, *p* < 0.05).

All the autologous, MHC-matched and MHC-mismatched recipients of MSC-primed showed a similar pattern of mild B cell response along all the time-points, except for a punctual increase in B cell proliferation induced by MHC-matched MSC-primed (T1 over: T0 and T5, *p* < 0.05) (Figure 7D).

For MSC-naïve (Figure 7E), both MHC-matched and MHC-mismatched groups showed similar proliferation rates for B cells after the first administration. However, after the second administration, an increase was observed in MHC-mismatched recipients (T4 over T1, *p* < 0.05; T5 over T0, *p* < 0.05; T6 over T0 and T2, *p* < 0.05), which was significant over the MHC-matched group (T4 and T6, *p* < 0.05). Nevertheless, at the end of the study, both MHC-matched and MHC-mismatched groups showed higher B cell response compared to the first administration (T7 over: T0, both groups, *p* < 0.05; T1, MHC-mismatched, *p* < 0.05; T2, *p* < 0.05, both groups; T3, MHC-mismatched, *p* < 0.01; T3, MHC-matched, *p* < 0.05).

### 3.2 Interferon gamma (IFNɣ) production as reflection of T cell activation: MSC-primed markedly induce IFNɣ secretion, whereas MSC-chondro promote minimal concentrations

**Figure 8:**
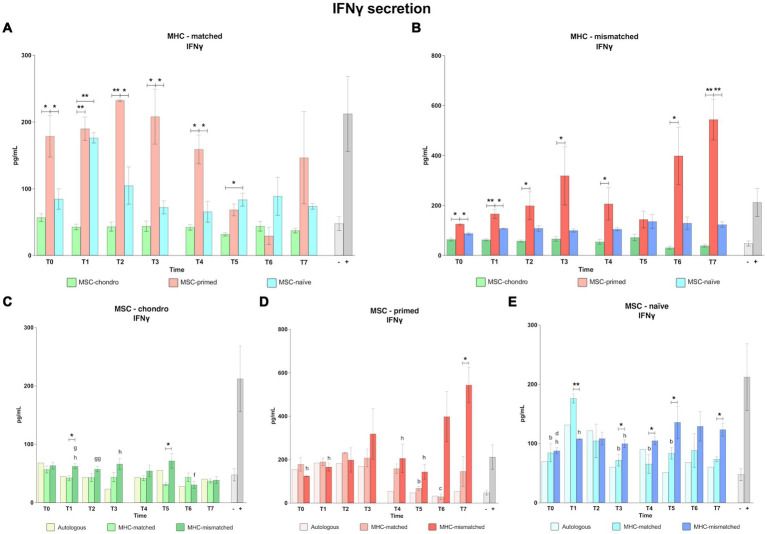
Concentration of interferon gamma (IFNɣ). Mean ± SEM concentration (pg/mL) of interferon gamma (IFNɣ) in co-culture supernatants from immunogenicity assays (modified one-way mixed lymphocyte reaction) in MHC-matched **(A)** and MHC-mismatched **(B)** recipients following the administration of MSC-chondro (green bars), MSC-primed (orange bars) and MSC-naïve (blue bars). Concentration of IFNɣ in the supernatant from autologous, MHC-matched and MHC-mismatched co-cultures exposed *in vitro* to MSC-chondro **(C)**, MSC-primed **(D)** and MSC-naïve **(E)**. Significant differences between cell-type and groups at one time-point are represented by a squared line with an asterisk (^*^p < 0.05 and ^**^p < 0.01). Significant differences between time-points are represented by lower case letter: T1, ^b^; T2, ^c^; T3, ^d^; T5, ^f^; T6, ^g^ and T7, ^h^ (^b^, ^c^, ^d^, ^f^, ^g^, ^h^, *p* < 0.05; ^gg^, *p* < 0.01).

The concentration of IFNγ was measured in the co-culture supernatants from all the modified one-way MLR assays as a reflection of T lymphocyte activation. Overall, MSC-primed, either MHC-matched or MHC-mismatched, induced more secretion of IFNγ than the other MSC types and than the positive control (classic MLR-mismatched) (Figure 8). Specifically, the IFNγ secretion induced by MSC-primed was significantly higher compared to MSC-chondro in both MHC-matched and MHC-mismatched co-cultures before and after the first administration (T0, *p* < 0.05 in both cases; T1, *p* < 0.01 in both cases; T2, MHC-matched, *p* < 0.01 and MHC-mismatched, *p* < 0.05; T3, *p* < 0.05 in both cases), and right before the second administration (T4, *p* < 0.05 in both cases) (Figures 8A,B). Moreover, after the second administration, MSC-primed MHC-mismatched also induced higher secretion of IFNγ over MSC-chondro (T6, *p* < 0.05; T7, *p* < 0.01) (Figure 8B).

The secretion of IFNγ induced by MSC-primed was also higher compared to MSC-naïve in both MHC-matched and mismatched settings, and both before (T0, *p* < 0.05) and after the first administration (MHC-matched: T2, T3 and T4, *p* < 0.05; MHC-mismatched: T1, *p* < 0.05) (Figures 8A,B). After the second administration, IFNγ induction by MSC-primed over MSC-naïve was overall higher in the MHC-mismatched group (T7, *p* < 0.01) (Figure 8B) but not in the MHC-matched group (Figure 8A). Although the IFNγ concentration detected in the MSC-naïve co-cultures was consistently higher than that observed for MSC-chondro, the difference between them was only significant in the MHC-matched co-cultures at 1 week after each administration (T1, *p* < 0.01; T5, *p* < 0.05) (Figure 8A).

When assessing the response to each cell type, MSC-chondro only induced mild secretion of IFNγ, but there were some interesting findings (Figure 8C). Levels of IFNγ were generally similar to the negative control (unstimulated PBLs alone) in the MHC-matched recipients along the time. However, in the MHC-mismatched group, IFNγ levels were higher than the negative control up to T5 and subsequently decreased (T6 and T7 over T1, *p* < 0.05; T6 over T2, *p* < 0.01; T6 over T5, *p* < 0.05; T7 over T3, *p* < 0.05). Moreover, IFNγ concentration in MHC-mismatched co-cultures was overall higher than in the MHC-matched ones (T1 and T5, *p* < 0.05).

Regarding MSC-primed (Figure 8D), IFNγ values were very similar between autologous and both allogenic MHC-matched/mismatched co-cultures after the first administration. However, after the second administration, IFNγ values significantly increased in the MHC-mismatched group (T7 over: T0, T1, T4 and T5, *p* < 0.05) while in the MHC-matched group IFNγ values decreased compared to the first administration (T5 over T1; T6 over T2, *p* < 0.05). Thus, IFNγ production after the second administration was consistently lower in the MHC-matched co-cultures, but significant differences over the MHC-mismatched co-cultures were only found at the end of the study (T7, *p* < 0.05).

For MSC-naïve (Figure 8E), even though MHC-mismatched co-cultures tended to induce more IFNγ, the highest IFNγ secretion was observed in MHC-matched co-cultures at 1 week after the first administration (T1 over: T0, T3, T4 and T5, *p* < 0.05; over MHC-mismatched group, *p* < 0.01). Aside from this observation, IFNγ secretion was higher in the MHC-mismatched co-cultures after the first administration (T3 over T0, *p* < 0.05; over MHC-matched group, *p* < 0.05) and it further increased after the second administration (T7 over T0, T1 and T3, *p* < 0.05; over T4, T5 and T7 MHC-matched group, *p* < 0.05).

## 4 Discussion

Previous studies have investigated the interaction of equine MSCs with lymphocytes *in vitro* (19, 49), but it is unclear whether *in vitro* findings translate into *in vivo* implications for therapy. Few *in vivo* works have been done in horses with this purpose, overall showing mild to moderate immune response (10, 50). However, the cellular immune response raised by MSCs has not usually been assessed comprehensively, and some factors, like MSC conditioning (priming, differentiation) or MHC compatibility, have been often overlooked. To the best of authors’ knowledge, this is the first study in the equine species assessing *in vivo* the systemic immune cellular response to allogenic MSCs taking into account, at the same time: (1) MSCs under different conditions (chondrogenically differentiated, pro-inflammatory primed, and basal), (2) the MHC compatibility, and (3) repeated administrations and serial evaluation. We performed a thorough assessment of the immune cellular response by covering the main subsets of lymphocytes (cytotoxic, helper and regulatory T cells, and B cells) and IFNγ secretion, by using a modified one-way MLR co-culture system previously validated *in vitro* (13, 17).

### 4.1 Main findings

We found a number of important observations: first, that equine MSCs under different conditions (priming, chondrogenic differentiation, basal) interact differently with the immune system *in vivo*. We hypothesized that MSCs primed with pro-inflammatory cytokines would be more efficient evading immune recognition while chondrogenically differentiated MSCs would be more easily recognized. In contrast, we observed that MSC-primed exhibited higher immunogenicity compared to MSC-chondro and MSC-naïve in terms of T cell response; however, MSC-primed also induced Treg and barely provoked a response by B cells, while the contrary was observed for MSC-chondro.

Secondly, this study shows that the compatibility for the MHC between donor and recipient is key in the systemic cellular immune response to equine MSCs *in vivo*. According to our initial hypothesis, MHC-mismatched MSCs of any type tended to induce higher T lymphocyte proliferation compared to MHC-matched MSCs; however, this response was not so marked for B cells. Of note, the response of T cell subsets was barely observed when analyzing their relative frequencies (Supplementary material S2), but it was clear when studying their proliferation rates. Specifically, MHC-mismatched recipients showed the greatest response for most of the T cell populations and at almost all time-points when receiving MSC-primed, and after the second administration of MSC-naïve. Additionally, although MSC-chondro induced the lowest effect on T cells, MHC-mismatched recipients of MSC-chondro showed greater changes in the cytotoxic response compared to MHC-matched ones. Previous studies *in vitro* also observed higher proliferation of T cells when exposed to MHC-mismatched equine MSCs that expressed high levels of MHC-II, in comparison to either MHC-matched MSCs (13) or MHC-mismatched MSCs but with low MHC-II expression (34). However, other works found that allogeneic MSCs (unknown MHC) were not inherently more immunogenic than autologous MSCs, as both types induced a similarly mild lymphocyte proliferation *in vitro* (33). This reinforces the notion that the results observed *in vitro* do not always accurately reflect the immune response that occurs *in vivo*, which leads to our third main finding.

We observed that cellular immune memory is generated *in vivo* by equine MSCs. This is based on the observation that equine lymphocytes responded more markedly to any type of MSCs in the one-way MLRs after the animals had received the MSCs, compared to the baseline (T0, prior to any MSC administration). This response varies along the time and, importantly, it is exacerbated by repeated administration of the same MSCs. However, interestingly, while MSC-primed generally tended to be more immunogenic, the cellular response after their second administration was similar to the first one. In contrast, the response elicited by MSC-naïve and MSC-chondro was further increased after a second administration. These findings also highlight the relevance of conducting *in vivo* studies to evaluate the immunogenicity of equine MSCs, and not to rely only on *in vitro* assessments using lymphocytes from animals that have not been exposed to MSCs. Moreover, evaluating only the frequencies of lymphocytes after MSC administration may not be enough to reveal the immune response, as we and others (17, 46) have found limited changes in the percentage of each lymphocyte subset while their proliferative response was marked and thus can provide further information.

### 4.2 Limitations of the study

Prior to engaging into more detailed discussion of our findings, it is important to acknowledge the limitations of this study. First, the sample size of animals is limited due to the challenges inherent to working with large animals like horses (51). Furthermore, finding animals that were matched for the MHC added additional complexity provided the high diversity of MHC haplotypes (35). The reduced sample size of the study could prevent extrapolating definitive recommendations to the general equine population, but considering the consistent trends observed for several of the analyses performed, our results can provide valuable insight into the cellular systemic immune response when administering equine MSCs. Second, the autologous group consisted of only three horses (one per MSC type), as they were involved solely for control purposes, similarly to previous works (40, 52). Third, the route of administration of MSCs (subcutaneous placement of hydrogels) does not accurately reflect clinical administration. Subcutaneous administration was chosen to make MSCs easily reached by the immune system as other locations (e.g., joints, eyes) may be less accessible, and the scaffold system was selected to immobilize the cells in a definite anatomic location and prevent their loss. In addition, this approach allowed recovering the scaffolds to be used in a separate study assessing the local response induced by the MSCs (unpublished data). However, the hydrogel could have also constituted a physical barrier for the interaction of MSCs and immune cells (41). Fourth, as aforementioned, the Treg subset could not be analyzed at some time-points due to supply issues of the anti-CD25 antibody, for reasons out of our control. Fifth, the possible interference of FBS antigens in the study cannot be completely disregarded, in spite of using the FBS removal strategy explained above that included exchanging FBS by autologous serum in the MSC culture for 24 h prior to administration, and several PBS washes. Moreover, to account for the possible effect of FBS antigens, or other unspecific antigens, we run several controls. It is worth noting that none of the negative controls (PBLs cultured alone and MLR-M) showed unspecific PBL proliferation, neither before administration nor at any time point of the study. Sixth, different methods for chondrogenic differentiation were used for the *in vivo* administrations and for the *in vitro* assays, thus potentially adding certain variability between the immune response produced *in vivo* and the proliferative response studied *in vitro*. The use of different protocols was needed to meet the requirements of this study for *in vivo* administration (MSCs embedded in an alginate hydrogel) and for *in vitro* cocultures (direct contact between MSC-chondro and PBLs, and control of the ratio between cell types). Moreover, to limit the possible interference of using different differentiation methods, we used MSC-chondro cells from the same donor and same passage in both assays and we previously compared the chondrogeneic outcome of both protocols (data not shown).

### 4.3 MSC-primed induce the strongest T cell response in MHC-mismatched recipients, but they may more immune-evasive than MSC-naïve and MSC-chondro after a second administration

According to our results, the systemic response of T cells against equine MSCs *in vivo* is influenced by the MHC compatibility and this response is dynamic, varying along the time after administration. The response of CD3^+^ lymphocytes also reflects how the immune recognition of MSCs *in vivo* depends on the pre-treatment done to the cells: MSC-naïve may be more quickly recognized after a second administration, while MSC-primed may initially evade this response, which might be attribute to a potentially higher regulatory capacity. While studying the general CD3^+^ T population provides important insight into the immune response (33, 53), dissecting T cell subsets reveals further differences.

We observed that equine MSCs can induce *in vivo* cytotoxic and helper T cell populations that are able to elicit a proliferative response when they encounter with the same MSCs again. In other words, cellular memory is generated against allogeneic equine MSCs. After the first administration, MSC-primed tended to induce a stronger CD8^+^ and CD4^+^ T cell response regardless of the MHC compatibility, while MSC-chondro and MSC-naïve did not seem to induce memory in the CD4^+^ population. Similarly, previous research (54) neither observed changes in the frequency of CD4^+^ population after *in vitro* re-exposure to MSCs at 120 days post single intra-articular injection of autologous or allogeneic equine MSC-naïve. However, lymphocyte proliferation was not assessed in that study, which could have revealed further changes (17). In contrast to the first administration, the second administration of MSC-chondro and MSC-naïve induced the response of both helper and cytotoxic T cells, but only in the MHC-mismatched group. However, interestingly, a second administration of MSC-primed did not increase the response of cytotoxic and helper T cells over the first administration. In spite of this, MSC-primed would still be the most immunogenic in our study based on T cell response. This increased immunogenicity could be attributed to the overexpression of MHC-I and MHC-II following MSC priming (13, 55). On the other hand, the enhanced immunomodulatory capacity of MSC-primed (19) could explain the slower and more moderate response of CD4^+^ and CD8^+^ T cells after their second administration.

To investigate deeper the response of T cells to equine MSCs, the supernatants from the *in vitro* co-cultures were analyzed to determine the presence of IFNγ, a pro-inflammatory cytokine produced by T lymphocytes that is associated with cell-mediated immunity. The secretion of IFNγ indicates the expansion of CD8^+^ or CD4^+^ effector and memory cells against donor MSCs (9). In agreement with the CD8^+^ and CD4^+^ T cell proliferative response, our results for IFNγ secretion also suggest that priming MSCs with pro-inflammatory cytokines can increase their immunogenicity, while MSC-chondro would produce the lowest T cell response. However, it cannot be completely disregarded the possible presence of residual exogenous IFNγ from the priming process, which might have interfered with the results.

Considering overall the T cell response, MHC-matched MSCs of any type prevented or moderated the proliferative and IFNγ production responses of helper and cytotoxic cells, especially after the second administration. The lower immunogenicity exhibited by MSC-chondro when administered in MHC-mismatched horses is consistent with previous reports using repeated administrations of allogeneic MSCs chondrogenically pre-differentiated in the horse (27). However, that study did not account for MHC compatibility, and our results show that MHC-mismatched MSC-chondro induced a significantly higher response than MHC-matched cells. Therefore, it remains to be determined whether the therapeutic effects of chondrogenically differentiated MSCs would be facilitated by MHC-matching. When administering MSC-naïve, the stronger cellular immune response after a second administration in MHC-mismatched recipients could have clinical implications, such as in terms of an adverse event (e.g., inflammatory response) and of potentially diminishing the therapeutic effects of MSCs because of their targeting for elimination. Thus, it would be advisable to consider using MHC-matched MSCs for repeated treatments. Selecting MHC-matched donors would be particularly important if cytokine priming strategies are used (1, 56) or if MSCs are to be injected in a highly inflammatory site, since we observed that MSCs exposed to pro-inflammatory cytokines may lead to an increased T cell response *in vivo* in MHC-mismatched recipients, even though the second administration of these cells might be better tolerated in terms of T cellular response.

### 4.4 MSC-primed, but not MSC-chondro, are able to induce a population of regulatory T cells *in vivo* that might help them modulating their immunogenicity

An increase in Treg would be related to enhanced immunosuppressive ability of MSCs, as this subset of lymphocytes would help in dampening the adaptive immune response and preventing rejection of foreign cells by the host (57). As for cytotoxic and helper responses, MSC-primed also produced the highest induction of regulatory T cells. Actually, the response along the time of Treg to MSC-primed in MHC-mismatched recipients suggests that the induction of this population may be a later event, which might be related with the more limited immunogenicity of MSC-primed after the second administration. Similarly, MHC-mismatched recipients of MSC-naïve showed the strongest Treg response steadily over time after the second administration, when the cytotoxic and helper responses were also higher. On the other hand, MSC-chondro, which had the mildest effect on CD8^+^ and CD4^+^ T cells, also presented a limited effect on the Treg subset. Therefore, equine MSCs may be eliciting an *in vivo* Treg response proportional to their immunogenicity, thus possibly suggesting a compensatory regulatory mechanism for the situations in which MSCs are more immunogenic (58). However, in the case of MSC-naïve, Treg induction would not limit the cytotoxic and helper response, and recipients of MSC-chondro did not show Treg induction corresponding to the time-points with an increased helper and cytotoxic response. Therefore, it might also be suggested that MSCs under different conditions would employ distinct mechanisms of immune evasion.

### 4.5 MSC-chondro, but not MSC-primed, induce a proliferative response of B lymphocytes *in vivo* regardless of MHC compatibility

The principal function of B lymphocytes is producing antibodies against foreign antigens (59). In contrast to Treg, MSC-primed produced minimal activation of B cells while MSC-chondro led to the most marked induction of this population. In agreement with our results, a previous study in rats (18) reported a significant increase in B cells when these were re-exposed *in vitro* to the chondrogenically differentiated MSCs that were firstly administered *in vivo*. On the contrary, the only *in vivo* study in the equine species that has investigated the capacity of chondrogenically induced allogeneic MSCs (ciMSCs) to activate B cells (CD138^+^ plasma cells), showed no response when re-exposing lymphocytes to ciMSCs *in vitro* (27). Of note, such co-cultures were carried out at variable times after administration (5 days to 1.5 years) and the MHC-haplotype was not examined.

While the type of MSCs influenced the response of B cells as also observed for T cells, the MHC compatibility did not seem to significantly affect their response, particularly in the case of MSC-chondro. The second administration of MSC-naïve and MSC-chondro produced an increase in B cell response, compared to the first exposure, in both MHC-matched and mismatched recipients. However, in MSC-chondro, this B cell induction was similar between MHC-matched and mismatched recipients. This could possibly indicate that chondrogenically differentiated equine MSCs might induce immune memory even in the absence of foreign MHC molecules. While we do not have a clear explanation for this finding, specific immunomodulatory mechanisms might be operating in MSC-chondro and/or the response of B cells might be mainly raised by antigens other than the MHC in differentiated cells. In addition, certain technical aspects of the one-way MLR system might have influenced the proliferation of B cells, such as the ratio MSC-chondro:PBLs, the potential presence of undifferentiated MSCs in the co-cultures, or the impact of TGF-β3 on MSC immunogenic profile. These considerations were also made in our earlier *in vitro* work (17), and their potential contribution to the present outcomes cannot be totally disregarded.

On the contrary, MSC-naïve produced a higher response in MHC-mismatched over MHC-matched recipients, but only after the second administration. First administration of MSC-naïve produced low proliferation rates for B cells, comparable to the baseline, in both MHC-matched and mismatched recipients, in agreement with previous studies (34). However, after repeated administration of MSC-naïve into MHC-mismatched animals, B cell proliferation progressively increased. Along with the T cell response observed, it might be suggested that MHC incompatibility is more critical for repeated administrations of MSC-naïve than for MSC-chondro and MSC-primed. This may suggest that the regulatory mechanisms of basal MSCs (MSC-naïve) may not be enough to evade the immune system, and thus manipulation prior to administration (differentiation, priming) may enhance their effects *in vivo*, as it has been suggested for treating equine joint pathologies (22, 27).

In contrast to MSC-naïve and MSC-chondro, MSC-primed showed the lowest B cell response consistently over the time and even after the second administration, suggesting that repeated administrations of MSC-primed would not induce B cell memory. Moreover, MSC-primed neither produced differences between MHC-matched and mismatched recipients. In agreement with our results, a previous *in vitro* study with human MSCs (60) observed that MSC-primed inhibited the proliferation of B cells over MSCs-naïve. These findings might be related with immune tolerance mediated by Treg, which were strongly induced by MSC-primed as discussed above. Therefore, it seems that MSC-primed may have unique immunomodulatory properties, in spite of their immunogenicity, that might prevent them from triggering a B cell immune response.

## 5 Conclusion

In summary, our results showed that MSC-chondro did not provoke a marked response of T lymphocytes, which could suggest that MSCs would not lose their regulatory ability neither would increase their immunogenicity after their chondrogenic differentiation. However, MSC-chondro induced proliferation of B cells and showed the lowest ability to stimulate Tregs, so they would not be able to completely evade the immune response. In contrast, MSC-primed promoted a higher and more sustained proliferation of T cells *in vivo*, but prevented the induction of B cells response, which might be an immunomodulatory mechanism mediated by Treg. Although MSC-naïve did not induce a marked cellular response after the first administration regardless of the MHC compatibility, the second administration of MHC-mismatched MSC-naïve provoked a sustained increase in helper, cytotoxic and B cell response compared to the first exposure and to the MHC-matched group.

In our conditions, and considering that this is an experimental study in a limited equine population, we may conclude that the type of MSC used and the degree of MHC compatibility can have a significant impact on the immune recognition of equine MSCs *in vivo*. Among the three types of MSCs analyzed, MSC-primed would induce the most marked immune response, followed by MSC-naïve, and lastly by MSC-chondro. However, the type of immune response can vary among the types of MSCs, possibly indicating different mechanisms of immune-evasion. The compatibility for the MHC would also be an important consideration for *in vivo* administration of equine MSCs, especially when using MSC-primed and when repeatedly administering MSC-naïve. For MSC-chondro, our findings suggest that MHC-mismatching might not be so critical, but the lower T cell response to MHC-matched MSC-chondro might be beneficial in a therapeutic setting. However, the induction of a B cell response by both MHC-matched and MHC-mismatched MSC-chondro should not be ignored due to the potential development of immunological memory.

The findings of this study can have important implications for the therapeutic use of MSCs and highlight the importance of strategies like the MHC selection of the MSC donor and of the culture conditions. Further investigation comprehensively addressing the immune response against equine allogeneic MSCs at various levels, including humoral and local immune responses, is crucial for the development of more effective and safer cell therapies for veterinary and human patients.

## Data availability statement

The raw data supporting the conclusions of this article will be made available by the authors, without undue reservation.

## Ethics statement

The animal study was approved by Advisory Ethics Committee for Animal Research from the University of Zaragoza (Project License PI 15/16). The study was conducted in accordance with the local legislation and institutional requirements.

## Author contributions

AC: Conceptualization, Data curation, Formal analysis, Methodology, Writing – original draft, Writing – review & editing. FV: Conceptualization, Methodology, Supervision, Writing – review & editing. AV: Conceptualization, Methodology, Writing – review & editing. EB: Conceptualization, Methodology, Writing – review & editing. SF: Conceptualization, Methodology, Writing – review & editing. MS: Conceptualization, Methodology, Writing – review & editing. MZ: Conceptualization, Funding acquisition, Supervision, Writing – review & editing. AR: Conceptualization, Methodology, Supervision, Writing – review & editing. CR: Conceptualization, Formal analysis, Funding acquisition, Project administration, Supervision, Writing – review & editing. LB: Conceptualization, Data curation, Formal analysis, Methodology, Supervision, Writing – review & editing.
