# Supplementary material for: The systemic cellular immune response against allogeneic mesenchymal stem cells is influenced by inflammation, differentiation and MHC compatibility: in vivo study in the horse
Source: Front Vet Sci. 2024 Jun 18;11:1391872. doi: 10.3389/fvets.2024.1391872 (PMC11217187; doi:10.3389/fvets.2024.1391872)
Supplement: Supplementary file 1 [file Supplementary_Material_1.MHC-haplotypes_horses.DOCX]

**Table S1.** List of MHC microsatellite haplotypes identified in the horses enrolled in the study.

|  | MHC class I | | MHC class III | | MHC class II | | | | | |  | |
| --- | --- | --- | --- | --- | --- | --- | --- | --- | --- | --- | --- | --- |
| Microsatellite  loci | UMNJH-38 | COR110 | ABGe9019 | UMNe65 | ABGe9030 | EQMHC 1 | COR112 | COR113 | UM011 | COR114 |  |  |
| Horses ID |  |  |  |  |  |  |  |  |  |  | **Haplotype** |  |
| **D1** | 165 | 221 | 301 | 261 | 215 | 190 | 262 | 270 | 179 | 241 | HapPRE10 |  |
|  | 165 | 221 | 301 | 261 | 215 | 190 | 262 | 270 | 179 | 241 | HapPRE10 |  |
| R1 | 165 | 221 | 301 | 261 | 215 | 190 | 262 | 270 | 179 | 241 | HapPRE10 |  |
|  | 156 | 215 | 301 | 261 | 215 | 190 | 262 | 270 | 179 | 241 | HapPRE10-like |  |
| R2 | 165 | 221 | 301 | 261 | 215 | 190 | 262 | 270 | 179 | 241 | HapPRE10 |  |
|  | 156 | 221 | 320 | 250 | 219 | 190 | 254 | 270 | 172 | 249 | HapMAI06 |  |
| R3 | 165 | 221 | 301 | 261 | 215 | 190 | 262 | 270 | 179 | 241 | HapPRE10 |  |
|  | 156 | 207 | 318 | 263 | 215 | 184 | 262 | 260 | 172 | 243 | HapPRE31 |  |
| X1 | 156 | 207 | 299 | 257 | 212 | 190 | 254 | 260 | 172 | 243 | HapMAI50 |  |
|  | 156 | 219 | 297 | 267 | 208 | 196 | 264 | 266 | 174 | 239 | Unknown1 |  |
| X2 | 163 | 207 | 312 | 261 | 211 | 192 | 262 | 268 | 176 | 247 | A3b |  |
|  | 161 | 211 | 305 | 259 | 215 | 184 | 252 | 280 | 172 | 247 | A3e-like |  |
| X3 | 156 | 221 | 307 | 255 | 211 | 192 | 254 | 266 | 167 | 249 | Unknown2 |  |
|  | 161 | 219 | 314 | 257 | 217 | 198 | 244 | 268 | 169 | 247 | Unknown3 |  |
| **D2** | 156 | 217 | 312 | 261 | 205 | 194 | 258 | 260 | 169 | 243 | HapMAI04 |  |
|  | 156 | 217 | 312 | 261 | 205 | 194 | 258 | 260 | 169 | 243 | HapMAI04 |  |
| A1 | 156 | 217 | 312 | 261 | 205 | 194 | 258 | 260 | 169 | 243 | HapMAI04 |  |
|  | 156 | 205 | 305 | 253 | 205 | 194 | 266 | 268 | 174 | 234 | HapPRE01 |  |
| A2 | 156 | 217 | 312 | 261 | 205 | 194 | 258 | 260 | 169 | 243 | HapMAI04 |  |
|  | 156 | 207 | 312 | 263 | 211 | 192 | 264 | 270 | 172 | 249 | Unknown4 |  |
| Y1 | 156 | 219 | 305 | 255 | 209 | 192 | 264 | 270 | 172 | 249 | HapPRE68 |  |
|  | 156 | 211 | 299 | 257 | 211 | 194 | 264 | 270 | 167 | 249 | HapPRA07 |  |
| Y2 | 156 | 205 | 318 | 263 | 215 | 190 | 248 | 270 | 172 | 236 | HapHA03 |  |
|  | 163 | 207 | 307 | 255 | 211 | 192 | 260 | 272 | 169 | 255 | HapPRA02 |  |
| Y3 | 156 | 219 | 316 | 261 | 205 | 194 | 260 | 274 | 172 | 247 | HapPSL08 |  |
|  | 156 | 219 | 316 | 261 | 205 | 194 | 260 | 274 | 172 | 247 | HapPSL08 |  |
| **D3** | 156 | 221 | 314 | 259 | 215 | 190 | 262 | 272 | 169 | 255 | HapPRE11 |  |
|  | 156 | 221 | 314 | 259 | 215 | 190 | 262 | 272 | 169 | 255 | HapPRE11 |  |
| C1 | 156 | 221 | 314 | 259 | 215 | 190 | 262 | 272 | 169 | 255 | HapPRE11 |  |
|  | 156 | 221 | 312 | 261 | 205 | 190 | 262 | 270 | 180 | 245 | Unknown5 |  |
| C2 | 156 | 221 | 314 | 259 | 215 | 190 | 262 | 272 | 169 | 255 | HapPRE11 |  |
|  | 156 | 211 | 301 | 259 | 209 | 192 | 262 | 268 | 174 | 234 | A2 |  |
| C3 | 156 | 221 | 314 | 259 | 215 | 190 | 262 | 272 | 169 | 255 | HapPRE11 |  |
|  | 156 | 207 | 314 | 261 | 215 | 190 | 262 | 270 | 180 | 247 | HapPRE26 |  |
| Z1 | 165 | 221 | 301 | 261 | 215 | 190 | 262 | 270 | 179 | 241 | HapPRE10 |  |
|  | 156 | 215 | 316 | 263 | 206 | 192 | 256 | 270 | 174 | 234 | Unknown6 |  |
| Z2 | | 156 | 217 | 307 | 255 | 205 | 194 | 258 | 270 | 180 | 245 | Unknown7 |
|  |  | 156 | 219 | 316 | 261 | 211 | 194 | 264 | 270 | 167 | 249 | Unknown8 |
| Z3 | | 156 | 219 | 314 | 259 | 207 | 192 | 268 | 274 | 180 | 245 | A8-like |
|  |  | 163 | 197 | 299 | 257 | 207 | 190 | 237 | 266 | 179 | 241 | A10b-like |

MHC homozygous horses are indicated in bold. Autologous, MHC-matched, and MHC-mismatched horses that received MSC-chondro (green), MSC-primed (orange) and MSC-naïve (blue).
