# Supplementary material for: The systemic cellular immune response against allogeneic mesenchymal stem cells is influenced by inflammation, differentiation and MHC compatibility: in vivo study in the horse
Source: Front Vet Sci. 2024 Jun 18;11:1391872. doi: 10.3389/fvets.2024.1391872 (PMC11217187; doi:10.3389/fvets.2024.1391872)
Supplement: Supplementary file 2 [file Supplementary_Material_2.Lymphocytes_frequencies.DOCX]

Supplementary Material

Changes in the frequency of lymphocytes subpopulations after in vitro re-exposure to MSCs (modified one-way MLR assay)

## CD3+ T Lymphocytes

The study evaluated the effect of different MSC types and MHC combinations on the activation of CD3+ T cells after two allogenic administrations. To this end, the percentage of CD3+ T cells was measured at different time-points following *in vitro* co-culture with the same MSCs administered *in vivo* (**Figure 1**). After the first administration, the percentage of CD3+ T lymphocytes remained similar under the different conditions, so neither the type of MHC combination nor the type of MSCs seemed to produce relevant changes in the global T cell population along the time. Throughout all the times analyzed, MSC-chondro decreased the percentage of CD3+ T cells compared to the negative control (classic MLR-matched), to MSC-naïve and to MSC-primed in both MHC-matched/mismatched combinations (**Figure 1A**, **1B**). Overall, MSC-primed tended to increase the percentage of CD3+ regardless of the type of combination along all the time-points. Specifically, in both MHC-matched and mismatched settings, MSC-primed increased the CD3+ population compared to MSC-chondro at T4 (*p <* 0.05; MHC-mismatched) (**Figure 1B)** and T5 (*p <* 0.05; MHC-matched and mismatched) (**Figure 1A**, **1B**). After the second administration at T5, MSC-primed MHC-matched also showed a significant increase compared to MSC-naïve (*p <* 0.05) (**Figure 1A**). After the second administration (T4, T5, T6 and T7), the percentage of T cells was significantly lower in MSC-chondro compared to MSC-naïve in the MHC-mismatched scenario (*p <* 0.05 in all cases) (**Figure 1B**). However, in the MHC-matched combination, MSC-chondro only promoted a significant reduction compared to MSC-naïve at T6 and T7 (*p <* 0.05 in both cases) (**Figure 1A**). Overall, mismatched combinations tended to further increase the percentage of T cells, but only MSC-chondro showed a significant increase (*p <* 0.05) compared to the matched setting at T7 (**Figure 1C**). Although, before second administration at T4, the mismatched MSC-chondro co-cultures reduced the percentage of CD3+ T cells compared to the MHC-matched group (*p <* 0.05), but not exceeding the control MLR-matched values (**Figure 1C**). Nevertheless, and in spite of finding some differences that statistically were significant, the changes in the frequency of the CD3+ population were very limited and may not be biologically relevant. On the contrary, the changes in the proliferative response of T cells that are shown in the main text, can better reflect the immune response against the different equine MSCs. This highlights the relevance of including the assessment of proliferation when MSC immunogenicity is evaluated, and to include the evaluation of more specific lymphocyte subsets.


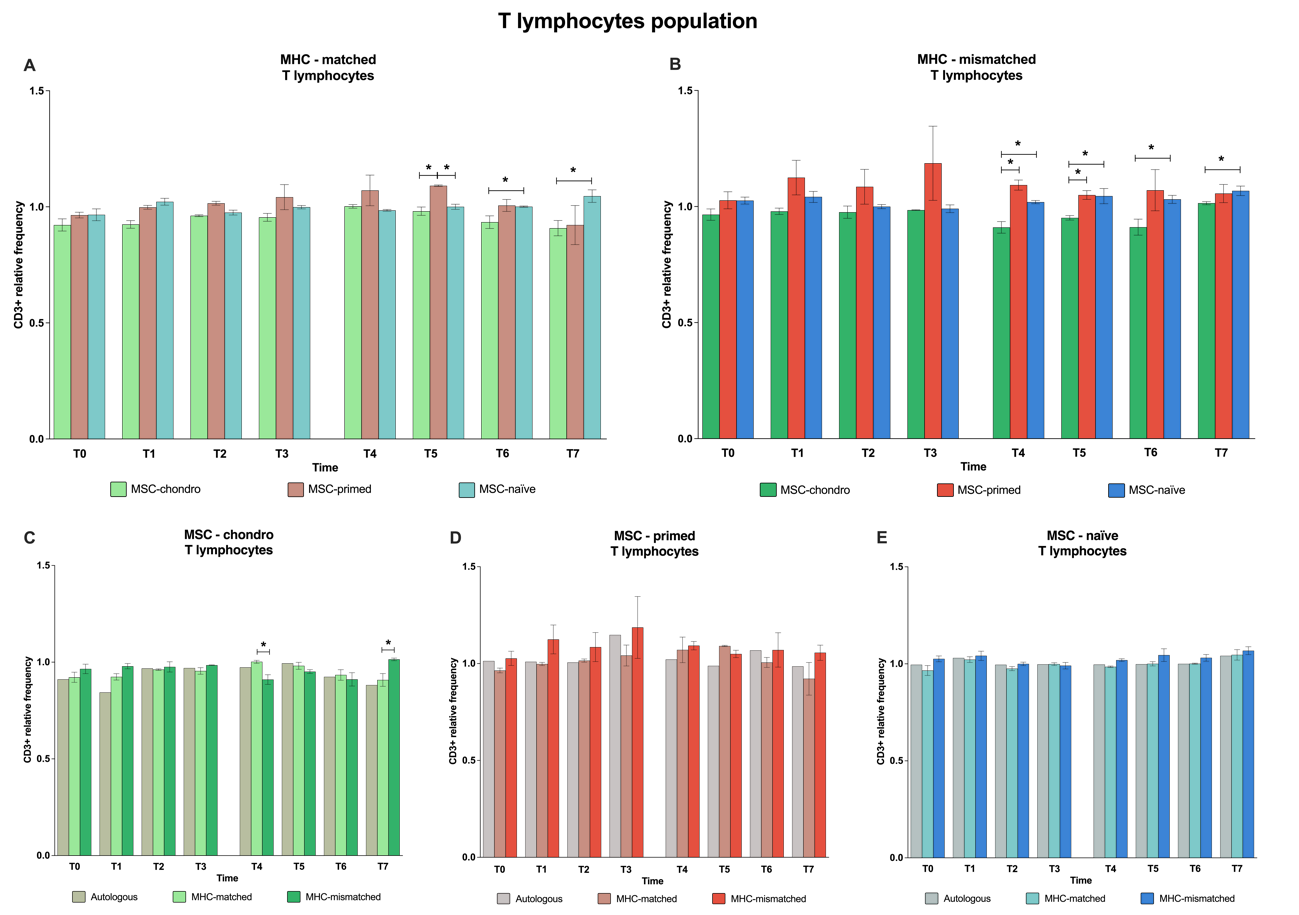


**Figure 1.** **Frequency of CD3+ T lymphocytes**. Mean ± SEM of the relative frequency of CD3+ T lymphocytes in the immunogenicity assays (modified one-way mixed lymphocyte reaction) in MHC-matched (**A**) and MHC-mismatched (**B**) recipients following the administration of MSC-chondro (green bars), MSC-primed (orange bars) and MSC-naïve (blue bars). Changes along time of non-activated PBLs from autologous, MHC-matched and MHC-mismatched recipients exposed *in vitro* to MSC-chondro (**C**), MSC-primed (**D**) and MSC-naïve (**E**). Data from each PBL recipient is normalized over the negative control (MLR M−, matched MLR) consisting of responder PBLs from the same donor exposed to MHC-matched stimulator PBLs (value 1), to account for inter-individual variability. Significant differences between cell-type and groups at one time-point are represented by a squared line with an asterisk (*, *p <* 0.05).

## CD8+ Cytotoxic T and CD4+ Helper T Lymphocytes

The present study also investigated the CD4+ T and CD8+ T cell responses along the time when administering allogeneic MSC-primed, MSC-chondro and MSC-naïve in MHC-matched or mismatched recipients. When recipients’ lymphocytes were re-exposed to the same MSCs *in vitro* (modified one-way MLRs), the percentage of CD8+ T cells tended to increase after the first administration of MSC-primed compared to MSC-chondro and MSC-naïve, for both MHC-matched and MHC-mismatched settings (**Figure 2**). These differences were statistically significant in MSC-primed compared to MSC-naïve from T1 to T3 for the MHC-mismatched setting (*p* < 0.05 in all cases) (**Figure 2B**). Interestingly, after re-exposure, MSC-chondro showed the highest increase in the percentage of CD8+ T cells for both allogenic co-cultures at T5 and T6, although the increase was statistically significant only for the MHC-matched co-culture at T5 over MSC-primed (*p* < 0.05) and MSC-naïve (*p* < 0.01) (**Figure 2A**). Moreover, this increase at T5 was statistically significant compared to T0 and T1 (*p* < 0.05 in both cases) in both MSC-chondro MHC-matched and MHC-mismatched co-cultures (**Figure 2C**).

Overall, MHC-mismatched combinations tended to increase the percentage of cytotoxic T cells compared to MHC-matched ones, but only MSC-primed showed a significant increase at T2 (*p* < 0.05) compared to the MHC-matched group (**Figure 2D**). Interestingly, while the CD8+ T cell population barely changed over time in the MSC-primed MHC-matched co-culture, a significant increase was observed in the MHC-mismatched co-cultures one week after the first administration (T1 over T0 and T4; *p* < 0.05 in both cases). This increase was also observed after the second administration, but it was not statistically significant until three weeks (T6 over T5; *p* < 0.05) (**Figure 2D**).


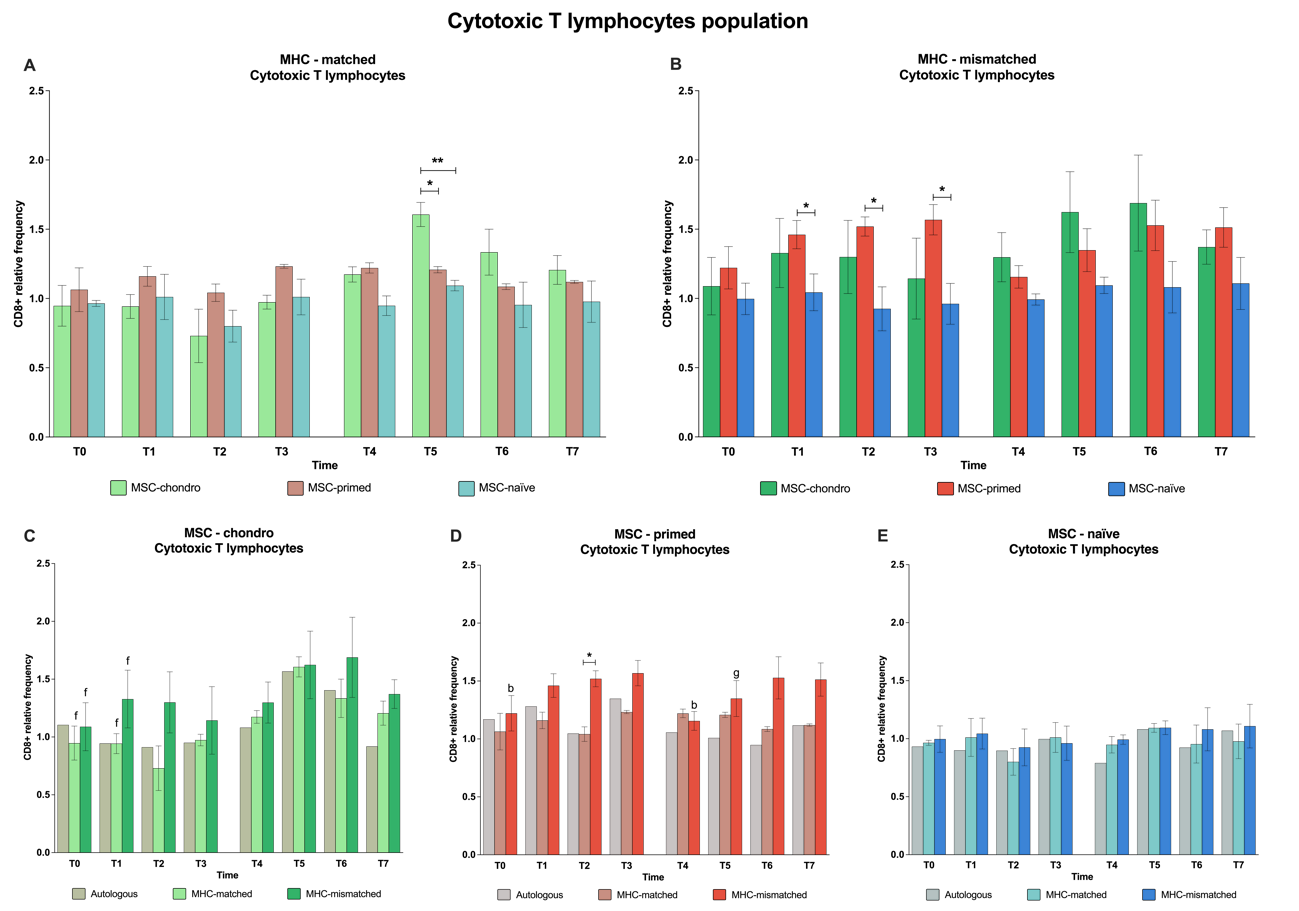


**Figure 2.** **Frequency of CD8+ cytotoxic T cells.** Mean ± SEM of the relative frequency of CD8+ cytotoxic T cells in the immunogenicity assays (modified one-way mixed lymphocyte reaction) in MHC-matched (**A**) and MHC-mismatched (**B**) recipients following the administration of MSC-chondro (green bars), MSC-primed (orange bars) and MSC-naïve (blue bars). Changes along time of non-activated PBLs from autologous, MHC-matched and MHC-mismatched recipients exposed *in vitro* to MSC-chondro (**C**), MSC-primed (**D**) and MSC-naïve (**E**). Data from each PBL recipient is normalized over the negative control (MLR M−, matched MLR) consisting of responder PBLs from the same donor exposed to MHC-matched stimulator PBLs (value 1), to account for inter-individual variability. Significant differences between cell-type and groups at one time-point are represented by a squared line with an asterisk (*, *p <* 0.05; ** *p <* 0.01). Significant differences between time-points are represented by lower case letter: T2, ^b^; T5, ^f^ and T6, ^g^ (^b^, ^f^, ^g^, *p <* 0.05).

In terms of CD4+ T cells, for the MHC-matched co-culture, both MSC-chondro and MSC-primed reduced the percentage of helper T cells compared to the MLR matched used as control along all the time-points, while MSC-naïve consistently showed a higher percentage of helper T cells over the other MSCs types (T3 and T6: *p* < 0.05 in all cases) (**Figure 3A**). In the MHC-mismatched groups, a similar trend of increasing the percentage of CD4+ T cells by MSC-naïve was observed over MSC-primed at T3 (*p* < 0.05) and over MSC-chondro at T0, T5, T6 and T7 (*p* < 0.05 in all cases) (**Figure 3B**).

In MSC-chondro, the CD4+ percentage increased in the MHC-mismatched co-cultures one week after each administration (T1 and T5). This percentage decreased at three weeks but increased again at 6 weeks after each administration (T3 and T7) compared to the other timepoints and to the MHC-matched co-cultures and the MLR-matched control (**Figure 3C**). Specifically, a significant increase in CD4+ percentage was observed at T3 over T1 and T4 (*p* < 0.05), but a significant decrease in helper T cells was observed at T6 compared to T5 (*p* < 0.05) (**Figure 3C**).

Similarly, MSC-primed MHC-mismatched co-cultures also promoted a significant increase in CD4+ T cells after the second administration at T6 and T7 compared to T2 and T3, respectively (*p <* 0.05 in both cases) (**Figure 3D**).

In MSC-naïve, a significant CD4+ T cells increase was observed after the first administration in both allogeneic groups (T3 compared to T1; *p* < 0.05 in both cases). In addition, MSC-naïve MHC-matched co-cultures induced a significant CD4+ increase at T6 compared to T2 (*p <* 0.05) (**Figure 3E**).

Regarding the effect of MHC-matched and MHC-mismatched combination, CD4+ and CD8+ T cells exhibited a similar trend, with higher levels induced by MHC-mismatched MSCs of any type. After the first administration, both MSC-primed and MSC-naïve MHC-mismatched co-cultures resulted in a significant increase in the percentage of helper T cells compared to the MHC-matched setting at T1 (*p* < 0.05 in both cases) (**Figure 3D**, **3E**), and at T3 for MSC-chondro (*p* < 0.05) (**Figure 3C**). After the second administration, a similar increase in CD4+ cells was observed in MHC-mismatched co-cultures at T5 for MSC-chondro and MSC-naïve (*p* < 0.05 in both cases) (**Figure 3C**, **3E**), and at T7 for MSC-primed (*p* < 0.05) (**Figure 3D**).


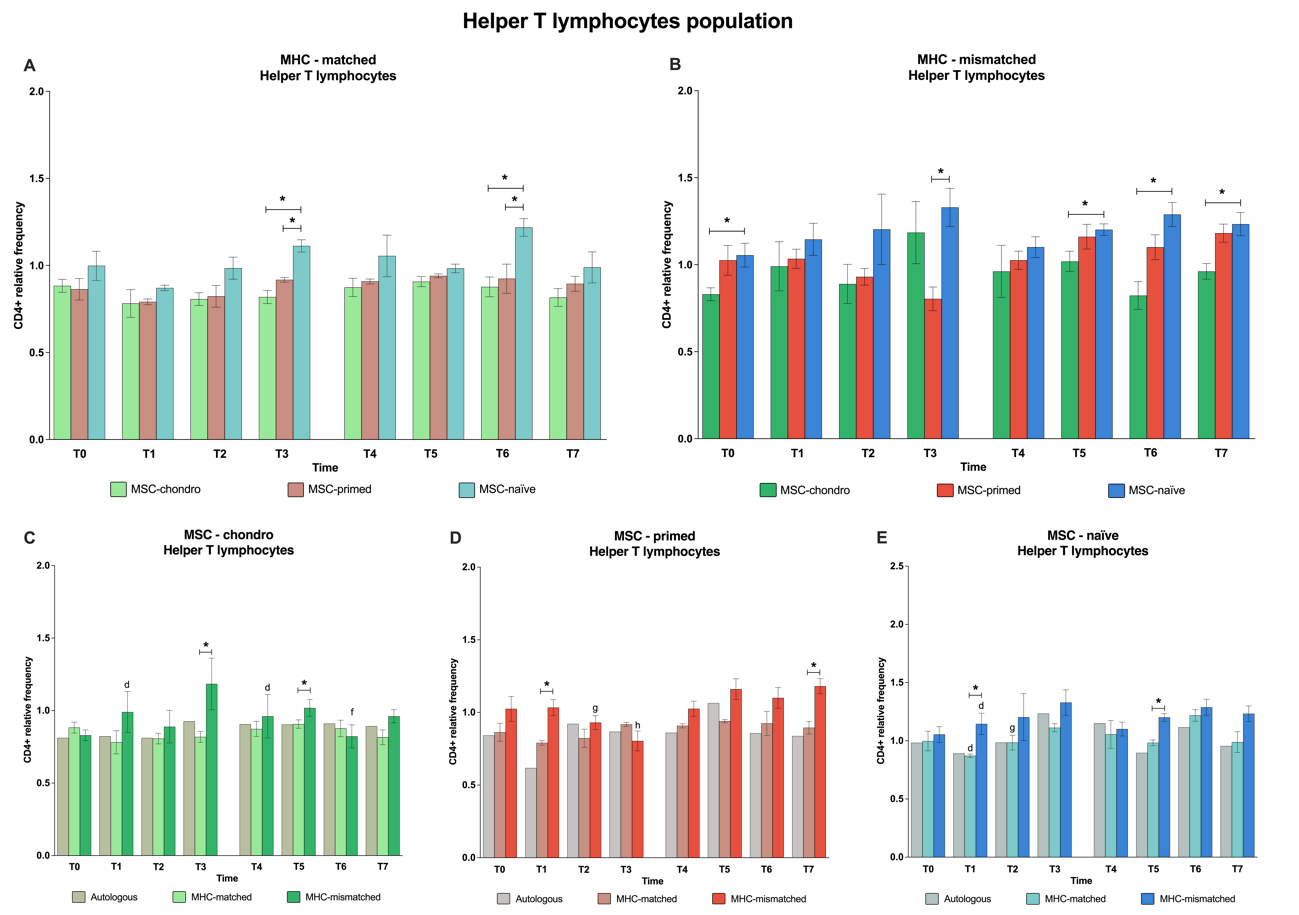


**Figure 3.** **Frequency of CD4+ helper T cells**. Mean ± SEM of the relative frequency of CD4+ helper T cells in the immunogenicity assays (modified one-way mixed lymphocyte reaction) in MHC-matched (**A**) and MHC-mismatched (**B**) recipients following the administration of MSC-chondro (green bars), MSC-primed (orange bars) and MSC-naïve (blue bars). Changes along time of non-activated PBLs from autologous, MHC-matched and MHC-mismatched recipients exposed *in vitro* to MSC-chondro (**C**), MSC-primed (**D**) and MSC-naïve (**E**). Data from each PBL recipient is normalized over the negative control (MLR M−, matched MLR) consisting of responder PBLs from the same donor exposed to MHC-matched stimulator PBLs (value 1), to account for inter-individual variability. Significant differences between cell-type and groups at one time-point are represented by a squared line with an asterisk (*, *p <* 0.05). Significant differences between time-points are represented by lower case letter: T3, ^d^; T5, ^f^; T6, ^g^ and T7, ^h^ (^d^, ^f^, ^g^, ^h^, *p <* 0.05).

## CD4+ CD25^high^ Regulatory T Cells

The effect of MSC type and MHC combination *in vivo* on the percentage of Treg was also analyzed in this study. MSC-primed was found to increase the percentage of Treg over MSC-naïve and MSC-chondro in all the conditions and time-points (**Figure 4A**, **4B**).

Comparison between MSC types showed that, before any administration (T0), allogenic MSC-primed already significantly increased Treg over MSC-chondro (*p* < 0.05 in both MHC-matched/mismatched). In addition, MSC-naïve MHC-mismatched co-cultures showed a Treg increase over MSC-chondro at T0 (*p* < 0.05) (**Figure 4A**, **4B**). It is noteworthy that the Treg increase observed for MSC-primed and MSC-naïve MHC-matched groups remained significant for all the time-points analyzed compared to MSC-chondro (MSC-primed: T3, T4, T5, T6, T7, *p* < 0.01; MSC-naïve: T3, T5 and T7, *p* < 0.01; T4 and T6, *p* < 0.05) (**Figure 4A**). For MHC-mismatched recipients, the same Treg patterns were observed in MSC-primed and MSC-naïve compared to MSC-chondro (MSC-primed: T1, T2, T3, T4, T5, T6 and T7; *p* < 0.01; MSC-naïve: T1, T5 and T7, *p* < 0.01; T6, *p* < 0.05) (**Figure 4B**). Interestingly, from T1 to T7, the percentage of Treg was always statistically higher in MSC-primed than in MSC-naïve for both allogenic MHC-matched (T3, T5 and T7, *p* < 0.05, T4 and T6, *p* < 0.01) and MHC-mismatched (T0 and T5, *p* < 0.05; T1, T2, T3, T4, T5, T6, and T7; *p* < 0.01) recipients (**Figure 4A**, **4B**).

The results showed that MSC-primed MHC-mismatched significantly increased the population of Treg over T0 at all the time-points analyzed (T1, T2, T3, T4, T5, T6, T7, *p <* 0.05) (**Figure 4D**). Furthermore, the Treg increase induced *in vivo* by MSC-primed MHC-mismatched was found to be significant at the latest time-points (T6 and T7) compared to T1, T4 and T5 (*p <* 0.05). Regarding the effect of donor-recipient compatibility, MHC-mismatched MSC-primed increased the Treg population over MHC-matched co-cultures at T0, T2, T6, and T7 (*p <* 0.05) (**Figure 4D**).

Regarding the effect induced by MSC-naïve on the Treg population, MHC-mismatched co-cultures tended to increase this population over MHC-matched (T0, *p* < 0.01; T3, *p* < 0.05) (**Figure 4E**). In the MHC-mismatched co-cultures, Treg cells increased steadily over time, particularly after the second administration, and a significant increase was observed at T5 and T7 compared to T0 (*p* < 0.05 in both cases) (**Figure 4E**). However, the opposite happened in MSC-naïve MHC-matched group, where the percentage of Treg was reduced over the baseline (T4, *p <* 0.05; T6, *p <* 0.01; T7, *p <* 0.05). In addition, significantly higher percentage of Tregs were observed at T3 compared to T7 in MSC-naïve MHC-matched co-cultures (*p <* 0.05) (**Figure 4E**).


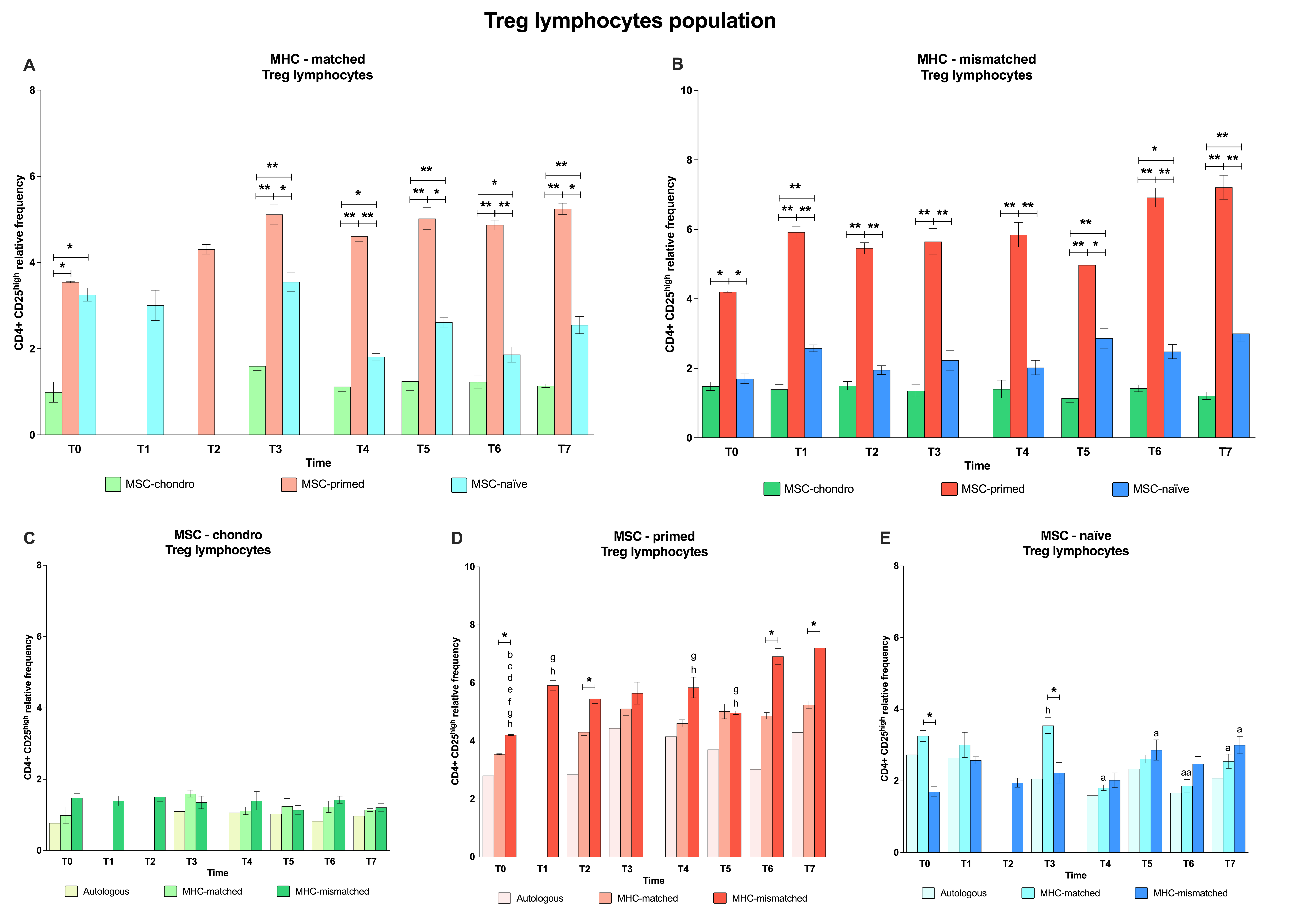


**Figure 4.** **Frequency of CD4+ CD25^high^ regulatory T cells.** Mean ± SEM of the relative frequency of CD4+ CD25^high^ regulatory T cells in the immunogenicity assays (modified one-way mixed lymphocyte reaction) in MHC-matched (**A**) and MHC-mismatched (**B**) recipients following the administration of MSC-chondro (green bars), MSC-primed (orange bars) and MSC-naïve (blue bars). Changes along time of non-activated PBLs from autologous, MHC-matched and MHC-mismatched recipients exposed *in vitro* to MSC-chondro (**C**), MSC-primed (**D**) and MSC-naïve (**E**). Data from each PBL recipient is normalized over the negative control (MLR M−, matched MLR) consisting of responder PBLs from the same donor exposed to MHC-matched stimulator PBLs (value 1), to account for inter-individual variability. Significant differences between cell-type and groups at one time-point are represented by a squared line with an asterisk (*, *p <* 0.05; **, *p <* 0.01). Significant differences between time-points are represented by lower case letter: T0, ^a^; T1, ^b^; T2, ^c^; T3, ^d^; T4, ^e^; T5, ^f^; T6, ^g^ and T7, ^h^ (^a^, ^b^, ^c^, ^d^, ^e^, ^f^, ^g^, ^h^, *p <* 0.05; ^aa^, *p <* 0.01).

## CD3- Pan-Ig+ CD21+ B Cells

The study found that in both allogenic MHC combinations, MSC-chondro and MSC-naïve tended to increase the percentage of B cells over MSC-primed, especially after the second exposure (**Figure 5**). Such increase was statistically significant for MSC-chondro in MHC-matched combinations at T6 over MSC-primed (*p <* 0.05) (**Figure 5A**). In addition, in the MHC-mismatched co-cultures, both MSC-chondro and MSC-naïve resulted in an increase in B cells compared to MSC-primed from T4 to T7 (*p <* 0.05 in all cases) (**Figure 5B**).

Moreover, after re-exposure, an increase in B cells percentage was observed for MSC-chondro and MSC-naïve in both allogenic groups compared to the first exposure, but these differences were only statistically significant for MSC-chondro (**Figure 5C**, **5E**). Specifically, a statistically significant increase in B cells was observed at T5 and T7 compared to T0 for MSC-chondro MHC-mismatched co-cultures (*p <* 0.05 in both times) and at T6 over T0 and T2 (*p <* 0.05 in both cases) for MHC-matched co-cultures (**Figure 5C**). On the other hand, for MSC-naïve, although there were no significant differences after the second administration, the frequency of B cells increased after the first administration at T3 over T0 in both MHC-matched (*p <* 0.05) and MHC-mismatched (*p <* 0.01) co-cultures (**Figure 5E**). Overall, the study found that mismatched combinations tended to further increase the percentage of B cells over time, although donor-recipient MHC matching or mismatching did not seem to have a significant effect, as observed for other lymphocyte subsets.


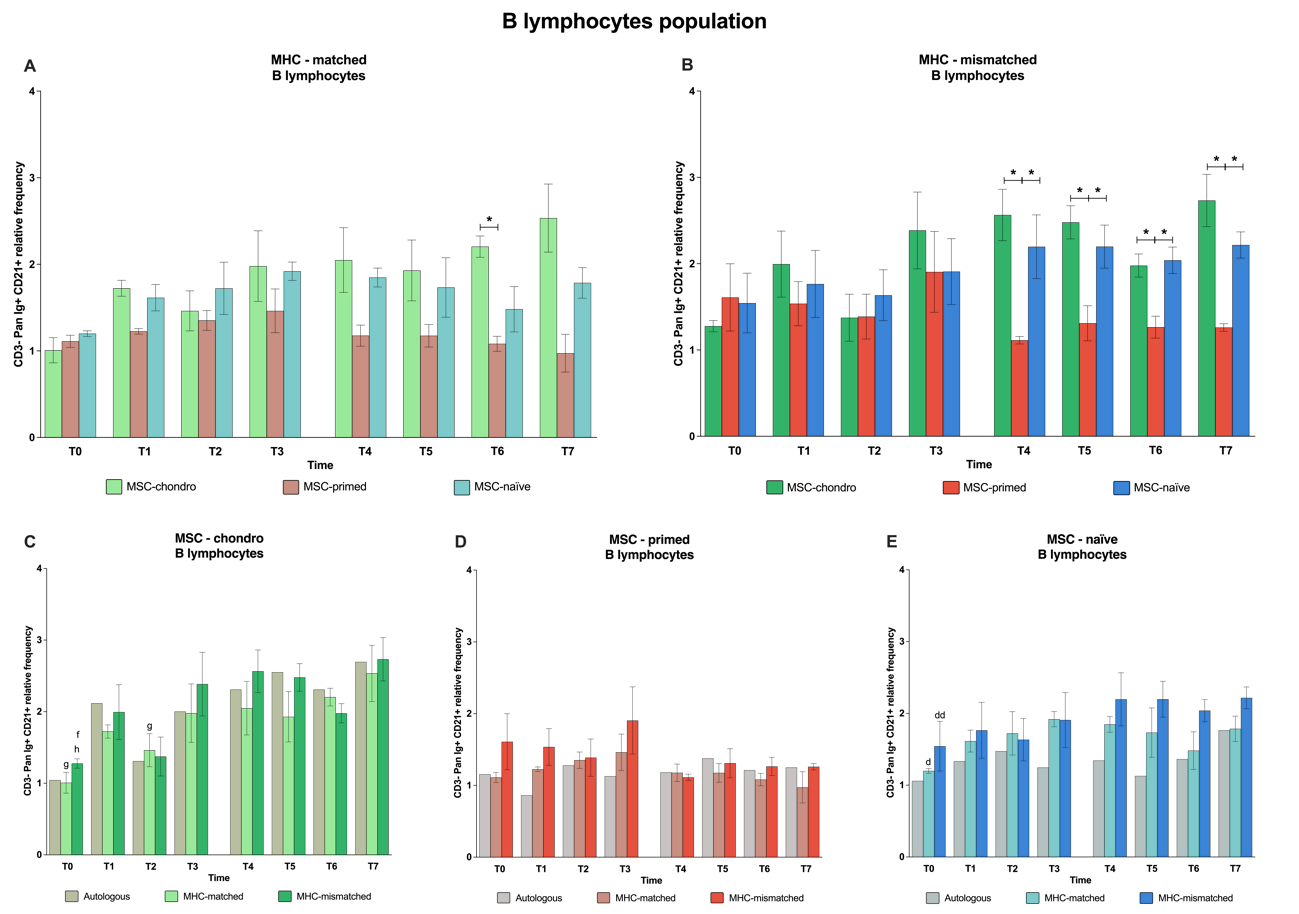


**Figure 5.** **Frequency of CD3−-Pan-Ig+ CD21+ B cells**. Mean ± SEM of the relative frequency of CD3−-Pan-Ig+ CD21+ B cells in the immunogenicity assays (modified one-way mixed lymphocyte reaction) in MHC-matched (**A**) and MHC-mismatched (**B**) recipients following the administration of MSC-chondro (green bars), MSC-primed (orange bars) and MSC-naïve (blue bars). Changes along time of non-activated PBLs from autologous, MHC-matched and MHC-mismatched recipients exposed *in vitro* to MSC-chondro (**C**), MSC-primed (**D**) and MSC-naïve (**E**). Data from each PBL recipient is normalized over the negative control (MLR M−, matched MLR) consisting of responder PBLs from the same donor exposed to MHC-matched stimulator PBLs (value 1), to account for inter-individual variability. Significant differences between cell-type at one time-point are represented by a squared line with an asterisk (*, *p <* 0.05). Significant differences between time-points are represented by lower case letter: T3, ^d^; T5, ^f^; T6, ^g^ and T7, ^h^ (^d^, ^f^, ^g^, ^h^, *p <* 0.05; ^dd^, *p <* 0.01).
